# Epidemiological shift and geographical heterogeneity in the burden of leptospirosis in China

**DOI:** 10.1186/s40249-018-0435-2

**Published:** 2018-05-18

**Authors:** Pandji Wibawa Dhewantara, Abdullah A. Mamun, Wen-Yi Zhang, Wen-Wu Yin, Fan Ding, Danhuai Guo, Wenbiao Hu, Federico Costa, Albert Icksang Ko, Ricardo J. Soares Magalhães

**Affiliations:** 1 Spatial Epidemiology Laboratory, School of Veterinary Science, The University of Queensland, Gatton, QLD 4343 Australia; 2 National Institute of Health Research and Development (NIHRD), Ministry of Health of Indonesia, Unit of Vector-borne Diseases Control, Pangandaran, West Java 46396 Indonesia; 3 Institute for Social Science Research, The University of Queensland, Indooroopilly, QLD 4068 Australia; 4 Center for Disease Surveillance and Research, Institute of Disease Control and Prevention of PLA, Beijing, 100071 People’s Republic of China; 5 Chinese Center for Disease Control and Prevention, Beijing, 102206 People’s Republic of China; 6 Scientific Data Center, Computer Network Information Center, Chinese Academy of Sciences, Beijing, 100190 People’s Republic of China; 7 School of Public Health and Social Work, Queensland University of Technology, Kelvin Grove, QLD 4059 Australia; 8 Instituto Gonçalo Moniz, Fundação Oswaldo Cruz, Ministério da Saúde, Salvador, BA 40296-710 Brazil; 9 Instituto da Saúde Coletiva, Federal University of Bahia (UFBA), Salvador, BA 40110-040 Brazil; 10 Department of Epidemiology of Microbial Diseases, Yale School of Public Health, New Haven, CT 06520 USA; 11 Children’s Health and Environment Program, Child Health Research Centre, The University of Queensland, South Brisbane, QLD 4101 Australia

**Keywords:** Leptospirosis, Epidemiology, Burden, China, DALY, Spatiotemporal trends

## Abstract

**Background:**

Leptospirosis morbidity and mortality rates in China have decreased since the 2000s. Further analyses of the spatiotemporal and demographic changes occurring in the last decade and its implication on estimates of disease burden are required to inform intervention strategies. In this study, we quantified the epidemiological shift and geographical heterogeneity in the burden of leptospirosis during 2005–2015 in China.

**Methods:**

We used reported leptospirosis case data from 1st January 2005 to 31st of December 2015 that routinely collected by the China Information System for Disease Control and Prevention (CISDCP) to analyze the epidemiological trend and estimate the burden in terms of disability-adjusted life-years (DALYs) over space, time, and demographical groups.

**Results:**

A total of 7763 cases were reported during 2005–2015. Of which, 2403 (31%) cases were the laboratory-confirmed case. Since 2005, the notified incidence rate was gradually decreased (*P* < 0.05) and it was relatively stable during 2011–2015 (*P* > 0.05). During 2005–2015, we estimated a total of 10 313 DALYs were lost due to leptospirosis comprising a total of 1804 years-lived with disability (YLDs) and 8509 years-life lost (YLLs). Males had the highest burden of disease (7149 DALYs) compared to females (3164 DALYs). The highest burden estimate was attributed to younger individuals aged 10–19 years who lived in southern provinces of China. During 2005–2015, this age group contributed to approximately 3078 DALYs corresponding to 30% of the total DALYs lost in China. Yet, our analysis indicated a declining trend in burden estimates (*P* < 0.001) since 2005 and remained relatively low during 2011–2015. Low burden estimates have been identified in the endemic regions where infections principally distributed. Most of the changes in DALY estimates were driven by changes in YLLs.

**Conclusions:**

In the last 11-years, the burden estimates of leptospirosis have shown a declining trend across the country; however, leptospirosis should not be neglected as it remains an important zoonotic disease and potentially affecting the young and productive population in economically less-developed provinces in southern of China. In addition, while in the last five years the incidence has been reported at very low-level, this might not reflect the true incidence of leptospirosis. Strengthened surveillance in the endemic regions is, hence, substantially required to capture the actual prevalence to better control leptospirosis in China.

**Electronic supplementary material:**

The online version of this article (10.1186/s40249-018-0435-2) contains supplementary material, which is available to authorized users.

## Multilingual abstract

Please see Additional file 1 for translations of the abstract into five official working languages of the United Nations.

## Background

Leptospirosis is a zoonotic disease of global public health importance caused by pathogenic spirochetes belong to the genus *Leptospira* [1]. It has caused more than one million cases and 58 900 deaths per year [2], and it has been estimated approximately 2.90 million disability-adjusted life-years (DALYs) lost due to leptospirosis worldwide [3]. Leptospirosis is acquired mainly through contact with contaminated water or soil containing *Leptospira*. In some cases, infections may also occur through direct contact with infected animals [4]. Due to non-specific clinical characteristics, leptospirosis often challenging to diagnose leading to underreported incidence which in turn may limit the effectiveness of control programme [5, 6].

Leptospirosis is an important zoonotic disease in China. It was firstly reported in 1934 and it becomes a mandatory notifiable disease since 1955. To date, there were more than 2.5 million cases and 20 000 deaths have been reported; more than 80% of the total provinces (34 provinces) have reported leptospirosis cases [7, 8]. Leptospirosis remains a significant public health problem in the country where a broad range of potential reservoirs and serovars are still circulating in the country. Furthermore, rapid population growth, poverty, and industrialization have led to excessive urbanization and environmental changes such as deforestation and urban expansion [9–13], which might influence disease nidality through direct impacts on the natural habitat of reservoirs and affects the spillover of infection between wildlife animals, domestic animals, and humans. Also, extreme weather events such as flooding following typhoons may significantly impact impoverish communities with lack of access to safe water and sanitation and health services [6], leading to an increased risk of *Leptospira* exposures.

Using notified leptospirosis morbidity and mortality data from the 1970s, a study has estimated that there were approximately 301 688 DALYs lost annually due to leptospirosis in China [3]. Nevertheless, in the last two decades, leptospirosis incidence has been reported to reduce from 10.73 cases per 100 000 people in the 1960s to 0.59 cases per 100 000 people in the 2000s [8, 10]. In the light of changes in socioeconomic and environmental conditions that have been undergone in China for the last two decades, there is a need to re-estimate the burden of leptospirosis and to identify residual pockets of transmission. To date, there is no single study have estimated the changes in burden in terms of DALYs across China over time.

Using available passive surveillance data on human leptospirosis in China, we aimed to investigate the changes on notified morbidity and mortality of leptospirosis and to quantify the demographical, temporal and geographical heterogeneity of the burden during 2005–2015. The findings of this study provide evidence to inform policy to allocate effective targeted intervention strategies for better leptospirosis control programmes in China.

## Methods

### Data sources

In China, leptospirosis is one of 39 notifiable infectious diseases that must be reported within 24-h (Category B disease) [14]. To illustrate, infectious diseases surveillance data, including leptospirosis, is analyzed by the Center for Disease Control and Prevention (CDC) at various level: county, prefecture, provincial, and national level. Case information is entered by all healthcare providers at all level via a nationally-standard form into a web-based Notifiable Infectious Diseases Reporting Information System (NIDRIS). In addition, a national system called China Infectious Disease Automated-alert and Response System (CIDARS) have been developed since 2005 to provide real-time outbreak notifications [15, 16]. In term of diagnosis, the provincial branches are responsible for testing suspected human patient and animal sera, collecting infected animals and identifying infectious isolates by culture and microscopic agglutination test (MAT) according to the national diagnostic criteria for leptospirosis issued by the National Health and Family Planning Commission (NHFPC) [17]. Results are then verified by the national-level CDC and finally reported to NHFPC.

In this study, we used reported leptospirosis case data from 1st January 2005 to 31st of December 2015. These data included information about age, gender, occupation, date of onset of illness, diagnosis and death, place of residence (i.e., county and province) and case classification (suspect, clinical, and confirmed). Yearly demographic data including population data by age, sex, and occupation was collected from the National Bureau of Statistics of China for each province from 2005 to 2015 [18].

### Human leptospirosis case definition

Based on NHFPC diagnostic criteria for leptospirosis [17], leptospirosis cases are defined into three categories: suspected, clinical, and confirmed case. Suspected cases are defined as an individual with: a) one of the following clinical symptoms such as acute fever (up to 39 °C) which may be accompanied by chills, myalgia, or malaise and; b) history of exposure within a month prior to the onset of illness to the following risk factors: epidemic season, reside in epidemic area, either direct or indirectly contacted with suspected animals and their urine or faeces or contaminated water and soil. Clinical (probable) cases are defined as suspected cases with at least one of the following clinical manifestations: conjunctival hyperemia, gastrocnemius tenderness, or enlargement of the lymph nodes. Confirmed case is defined as suspected case with one or more any of the following laboratory criteria: 1) positive culture of *Leptospires* from blood, urine, tissues, or cerebrospinal fluid (CSF); 2) Microscopic Agglutination Test (MAT) titre of ≥400 in single or paired serum samples; 3) a fourfold or greater rise in MAT titers between acute and convalescent-phase samples; 4) presence of pathogenic *Leptospira* spp. detected by polymerase chain reaction (PCR); 5) presence of IgM antibodies by enzyme-linked immunosorbent assay (ELISA).

### Data analysis

For our analyses, we divided China into four regions (Region A, B, C, and D) as previously described by Zhang et al. [7] and investigated the temporal distribution of notified leptospirosis incidence and mortality across the four regions. The proportion of deaths (case-fatality-rates, CFR) due to leptospirosis infection was calculated. As we observed that there were different trends during 2005–2015, we then divided the dataset into two temporal subgroups: 2005–2010 and 2011–2015 to investigate epidemiological changes between these two periods. We used yearly population data as the denominator to calculate age-, gender-, and occupation-specific incidence and mortality rates over time.

A temporal analysis of morbidity and mortality of leptospirosis was conducted by gender, age groups, occupation, regions and case classification. We classified occupational group into three main categories based on the type of industry: primary, secondary, and tertiary workforces. Farmers, plant growers, herdsman, seaman, and fishers were categorized as primary (agricultural-related) workforces. The secondary industry was defined as manufacture-related work. The tertiary workforce was defined for those individuals who work in services (e.g., teachers, doctors, nurses, students). The occupation classified as “others” include individuals who retired/not working, including children and undefined profession.

A simple linear regression model was used to detect a trend in reported incidence, mortality, and burden estimates during 2005–2015, with the independent variable being the year and the dependent variable being a number of the case or disease rate. A chi-square (χ^2^) test was performed to determine the difference in incidence, mortality rate and burden by age, sex, and occupation in different time periods. *P-*value < 0.05 were considered statistically significant. A multiplicative seasonal decomposition analysis was conducted using SPSS version 24 (IBM Corp., Armonk, NY, USA) to examine seasonality of leptospirosis incidence. Changes in the spatial distribution of incidence and burden during two periods were mapped using ArcGIS 10.5 (ESRI Inc., Redlands, CA, USA). All other statistical analysis was conducted using STATA version 13.0 (Stata Corp., College Station, TX, USA).

### DALYs estimation

Based on total cases, we estimated the burden in terms of age-, sex- and province-specific DALYs during the period of study. We estimated the DALYs for each year by adding the number of Years of Life Lost due to death in the population (YLLs) and the number of Years Lived with Disability (YLDs) due to the disease [19]. The estimate of YLLs was obtained by multiplying number of cases per year and the standard life expectancy at the age of death in years. To estimate life expectancy at the age of death, we used a standard life table used for the estimation of Global Burden of Disease 2010 [20]. YLDs were calculated by multiplying incidence, disability weight (DW), and duration of the illness. The disability weight used for the estimation of YLDs was the same that used in a study elsewhere [3]. In brief, all death cases were defined as fatal cases. Thus, we were given a DW of 0.573 for one month as it was assumed that they had dialysis before death. For non-fatal cases, we assumed that there were 70% acute cases and 30% had chronic sequelae. Of those acute cases, 50% is mild (given a DW 0.053 for 2 months), 40% is moderate (DW 0.21–2 months), and 10% severe (DW 0.562 for 2 weeks, 0.51 for 2 weeks, and 0.21 for 1 month). Of those chronic cases, a DW of 0.245 for two months to three years was given. In our DALYs calculation, we did not consider age-weighting and discounting.

## Results

### Descriptive analysis

A total of 7763 leptospirosis cases were reported during 2005–2015 (Table 1). Of these, 2403 cases (31%) were recorded as confirmed cases, 4588 (59%) of cases as clinical cases, and 772 (10%) cases were reported as suspected cases. The proportion of confirmed cases towards the total cases was increased over time from 8.2% (120/1465) in 2005 to 56.7% (233/411) in 2015. The proportion of reported laboratory-confirmed case during 2005–2015 was varied across 26 provinces in China (Additional file 2: Table S1).

**Table 1:** Annual number of notified leptospirosis and incidence rate by sex, age and occupation and the proportion of case in China, 2005–2015

Characteristics		Number of cases (per 100 000 people)											Total	IR^a^
		2005	2006	2007	2008	2009	2010	2011	2012	2013	2014	2015		
Sex	Female	439 (0.07)	217 (0.04)	300 (0.05)	254 (0.04)	197 (0.03)	218 (0.04)	134 (0.02)	154 (0.03)	156 (0.03)	207 (0.04)	131 (0.02)	2407	0.03
	Male	1026 (0.16)	500 (0.08)	658 (0.10)	675 (0.11)	462 (0.08)	500 (0.08)	289 (0.05)	337 (0.06)	280 (0.05)	349 (0.06)	280 (0.04)	5356	0.08
Age	0–9	41 (0.03)	28 (0.02)	34 (0.03)	20 (0.02)	18 (0.01)	10 (0.00)	8 (0.01)	4 (0.00)	5 (0.00)	6 (0.00)	3 (0.00)	177	0.01
	10–19	283 (0.13)	116 (0.06)	137 (0.07)	99 (0.06)	83 (0.05)	52 (0.03)	33 (0.02)	34 (0.03)	15 (0.01)	24 (0.02)	22 (0.01)	898	0.05
	20–29	236 (0.14)	86 (0.06)	107 (0.07)	120 (0.08)	70 (0.04)	62 (0.04)	40 (0.02)	47 (0.02)	48 (0.03)	71 (0.04)	45 (0.02)	932	0.05
	30–39	347 (0.14)	140 (0.07)	174 (0.09)	174 (0.09)	112 (0.06)	131 (0.07)	66 (0.04)	67 (0.04)	53 (0.03)	89 (0.05)	63 (0.03)	1416	0.07
	40–49	228 (0.12)	140 (0.07)	202 (0.10)	180 (0.09)	126 (0.06)	159 (0.08)	99 (0.05)	126 (0.06)	106 (0.05)	142 (0.07)	83 (0.03)	1591	0.07
	50–59	217 (0.15)	130 (0.08)	205 (0.12)	210 (0.12)	137 (0.08)	167 (0.10)	101 (0.08)	97 (0.07)	92 (0.07)	111 (0.08)	95 (0.05)	1562	0.09
	60–69	87 (0.09)	53 (0.06)	75 (0.08)	93 (0.10)	92 (0.09)	112 (0.12)	55 (0.06)	84 (0.09)	83 (0.09)	84 (0.09)	83 (0.06)	901	0.08
	70–79	16 (0.03)	15 (0.03)	23 (0.04)	30 (0.05)	20 (0.04)	27 (0.05)	20 (0.04)	32 (0.06)	23 (0.05)	22 (0.04)	25 (0.04)	253	0.04
	80–89	1 (0.00)	0.00	6 (0.04)	4 (0.02)	1 (0.00)	0.00	3 (0.02)	3 (0.02)	4 (0.02)	5 (0.03)	3 (0.01)	30	0.02
	90+	0.00	0.00	1 (0.07)	0.00	0.00	0.00	0.00	0.00	1 (0.05)	0.00	1 (0.05)	3	0.02
Occupation	Agriculture	1119 (0.32)	500 (0.15)	721 (0.22)	726 (0.24)	526 (0.18)	571 (0.22)	312 (0.11)	387 (0.14)	312 (0.12)	466 (0.19)	305 (0.13)	5944	0.18
	Services	263 (0.11)	125 (0.05)	148 (0.06)	110 (0.03)	90 (0.02)	63 (0.02)	54 (0.02)	39 (0.01)	54 (0.02)	56 (0.02)	56 (0.02)	1058	0.04
Case classification	Confirmed	120	227	267	249	158	229	178	182	239	321	233	2403	
	Clinical	1271	415	594	600	406	436	212	247	123	171	113	4588	
	Suspected	74	75	97	80	95	53	33	58	78	64	65	772	

^a^mean annual reported incidence rate

By gender, a total of 5356 (69%) males and 2407 (31%) females were observed. The annual incidence rate (IR) was higher in males (0.08 cases per 100 000 people) compared to females (0.03/100 000 people) (*χ*^2^ = 22.50, *P* = 0.013, Table 1). Our results indicate that incidence differed significantly by age group (*χ*^2^ = 624.57, *P* < 0.001) with most of the cases (21%) reported in the 40–49 years old age group. The higher incidence rate was identified in the older age groups of 50–59 (0.09/100 000 people) and 60–69 (0.08/100 000 people). However, leptospirosis in younger individuals under 20 years-old was also reported, which accounted for a total of 1075 cases (14%) with reported incidence rates ranging from 0.01–0.05/100 000 people. Based on patients’ occupation, we could only identify two main occupational type: primary (agriculture) and tertiary (services). Of 761 records were classified as ‘Other’ including who did not work, children, and retired people (Additional file 2: Table S2). Leptospirosis incidence rate was higher in primary sector (0.18/100 000 people) compared to the group of tertiary workforces (0.04/100 000 people) (*χ*^2^ = 47.15; *P* < 0.001). Furthermore, among the primary workforces, of 298 (5%) cases were attributed to young farmers aged under 20 years old. Whereas, among the tertiary workforces, most cases (43%) was predominantly attributed to students aged under 20 years.

**Fig. 1:**
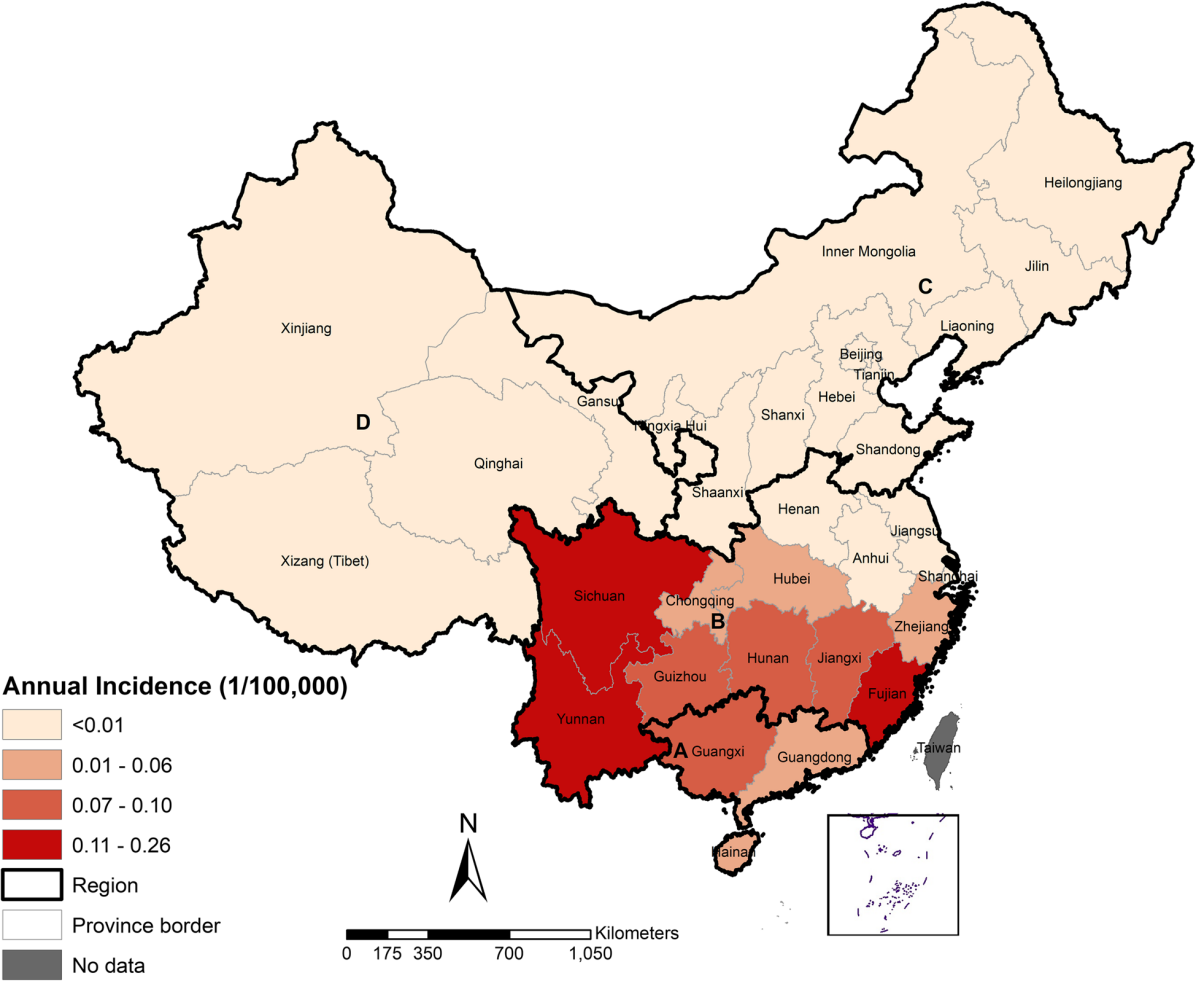
Annual average incidence map of leptospirosis in China, 2005–2015**.** It is divided into four regions (Region A, B, C, D) refer to Zhang et al. [7]

**Fig. 2:**
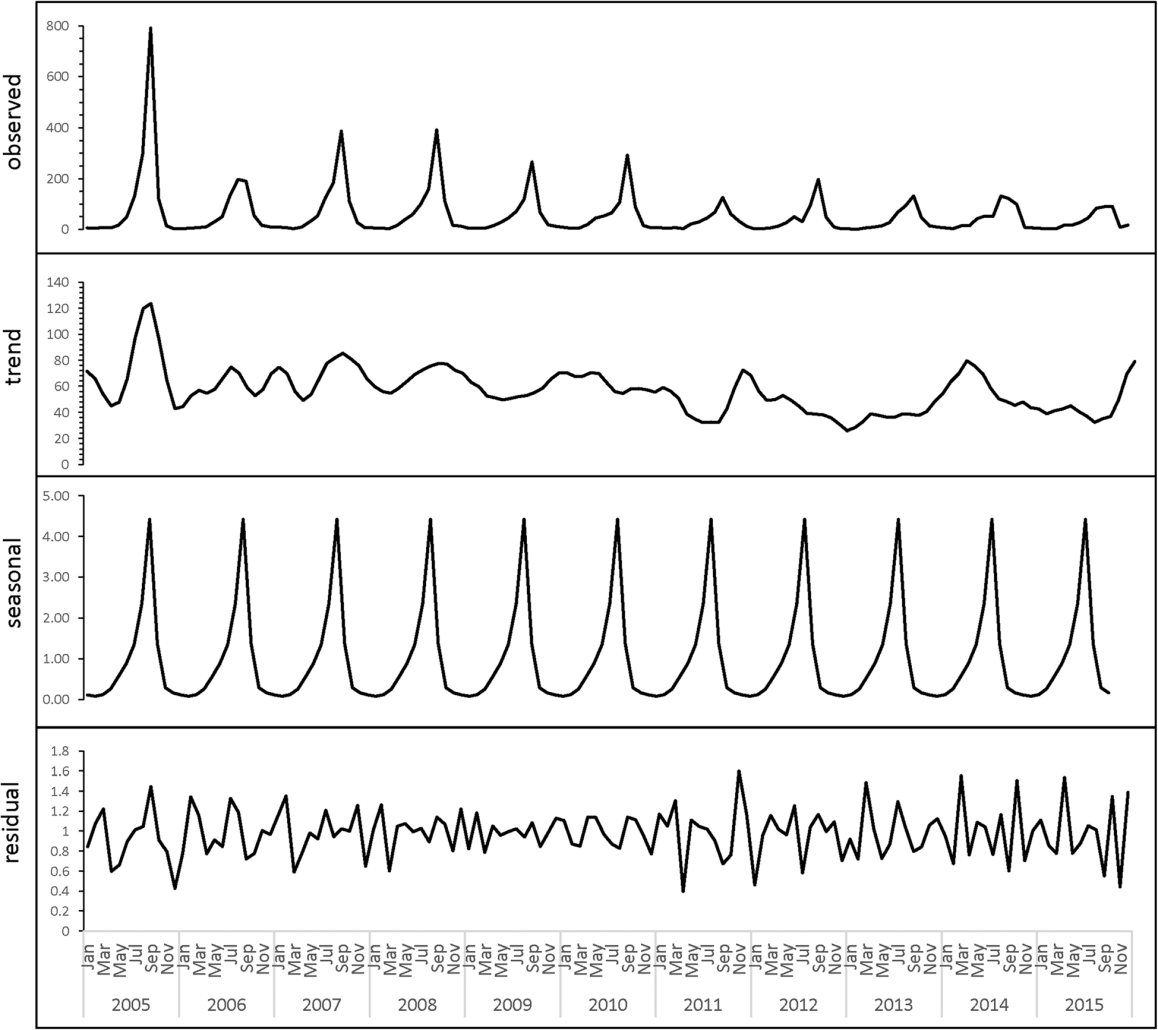
Seasonal decomposition plot of leptospirosis cases in China

Leptospirosis was reported from all four regions in China; the highest reported incidence rate was primarily observed in the Region A and B (Fig. 1). Based on the seasonal decomposition analysis, leptospirosis incidence increased from April to September and reached a peak on August/September and then diminished from October thereafter. The lowest number of cases was consistently observed in February (Fig. 2).

In total, leptospirosis cases were reported from a total of 782 counties in 26 provinces in China (Table 2). Most leptospirosis cases were reported from Region B, where 6514 cases (84%) were reported during 2005–2015. The number of counties that reported cases demonstrated a significant reduction in 2005–2015 (*P* < 0.001). Overall, there was a significant declining trend in leptospirosis incidence from 0.11/100 000 people in 2005 to 0.03/100 000 people in 2015 (R^2^ = 0.646; *P* < 0.05). Among provinces, both Sichuan and Yunnan had the highest reported incidence rate (0.26/100 000 people) in the country. Similarly, the case-fatality rates (CFR) also showed a downturn trend from 3.29% in 2005 to 0.24% in 2015 (R^2^ = 0.815, *P* < 0.001). The highest CFR was recorded in Guizhou (13.41%) compared to other provinces. Detailed temporal distribution of leptospirosis incidence and CFR for each province during 2005–2015 is provided (see Additional file 2: S1 Table, S3 Table).

**Table 2:** Annual reported cases, confirmed case, counties, incidence and fatality rate of leptospirosis in China, 2005–2015

	No. of cases reported (*n* = 7763)	% confirmed case (*n* = 2403)	No. of county reported (*n* = 782)	Incidence per 100 000 people	CFR (%)
Year					
2005	1465	8.2	307	0.11	3.29
2006	717	31.7	265	0.05	2.66
2007	958	27.9	299	0.07	3.86
2008	929	26.8	278	0.07	2.04
2009	659	24	222	0.05	1.82
2010	718	31.9	239	0.05	1.53
2011	423	42.1	182	0.03	1.17
2012	491	37.4	180	0.04	1.02
2013	436	54.3	165	0.03	1.16
2014	556	57.7	173	0.04	1.07
2015	411	56.7	163	0.03	0.24
Region A					
Guangdong	619	53.3	100	0.06	2.28
Guangxi	543	40.1	92	0.10	1.16
Hainan	47	10.6	15	0.05	0.00
Region B					
Jiangsu	37	54.1	25	0.00	1.30
Zhejiang	138	42.8	28	0.02	1.31
Anhui	310	8.4	31	0.05	1.03
Fujian	502	47	62	0.12	0.74
Jiangxi	421	5.5	52	0.09	1.62
Henan	3	0	3	0.00	0.00
Hubei	296	10.8	31	0.05	2.96
Hunan	656	15.9	98	0.09	3.10
Chongqing	200	10	33	0.06	0.49
Sichuan	2352	6.3	97	0.26	1.10
Guizhou	291	11.7	45	0.07	13.41
Yunnan	1308	86.2	36	0.26	0.15
Region C					
Beijing	2	50	2	0.00	0.00
Shandong	20	65	14	0.00	0.00
Hebei	3	33.3	3	0.00	0.00
Shanxi	3	33.3	3	0.00	0.00
Inner Mongolia	1	100	1	0.00	0.00
Liaoning	1	0	1	0.00	0.00
Jilin	2	100	2	0.00	0.00
Shaanxi	4	25	4	0.00	0.00
Region D					
Gansu	1	100	1	0.00	0.00
Qinghai	1	0	1	0.00	0.00
Xinjiang	2	0	2	0.00	0.00

In addition, during 2005–2015, a total of 168 deaths attributed to leptospirosis reported in China. Of which, 71% (120/168) of reported deaths were attributed to males. High mortality rate was observed attributed to group 50–59 years (0.20 per 100 000 people) followed by a group of age 10–19 years (0.18 per 100 000 people). A high number of death (125 out of 168) were attributed to patients who worked in the primary sector as a farmer. A high number of death reported from Region B (74%), particularly in Guizhou, Sichuan, Hunan, Hubei, and Jiangxi (Additional file 2: Table S4). There was a decrease in reported mortality rate since 2005 and it reached a very low level from 2011 thereafter.

### Demographic and geographical changes in morbidity and mortality between 2005 and 2010 and 2011–2015

**Fig. 3:**
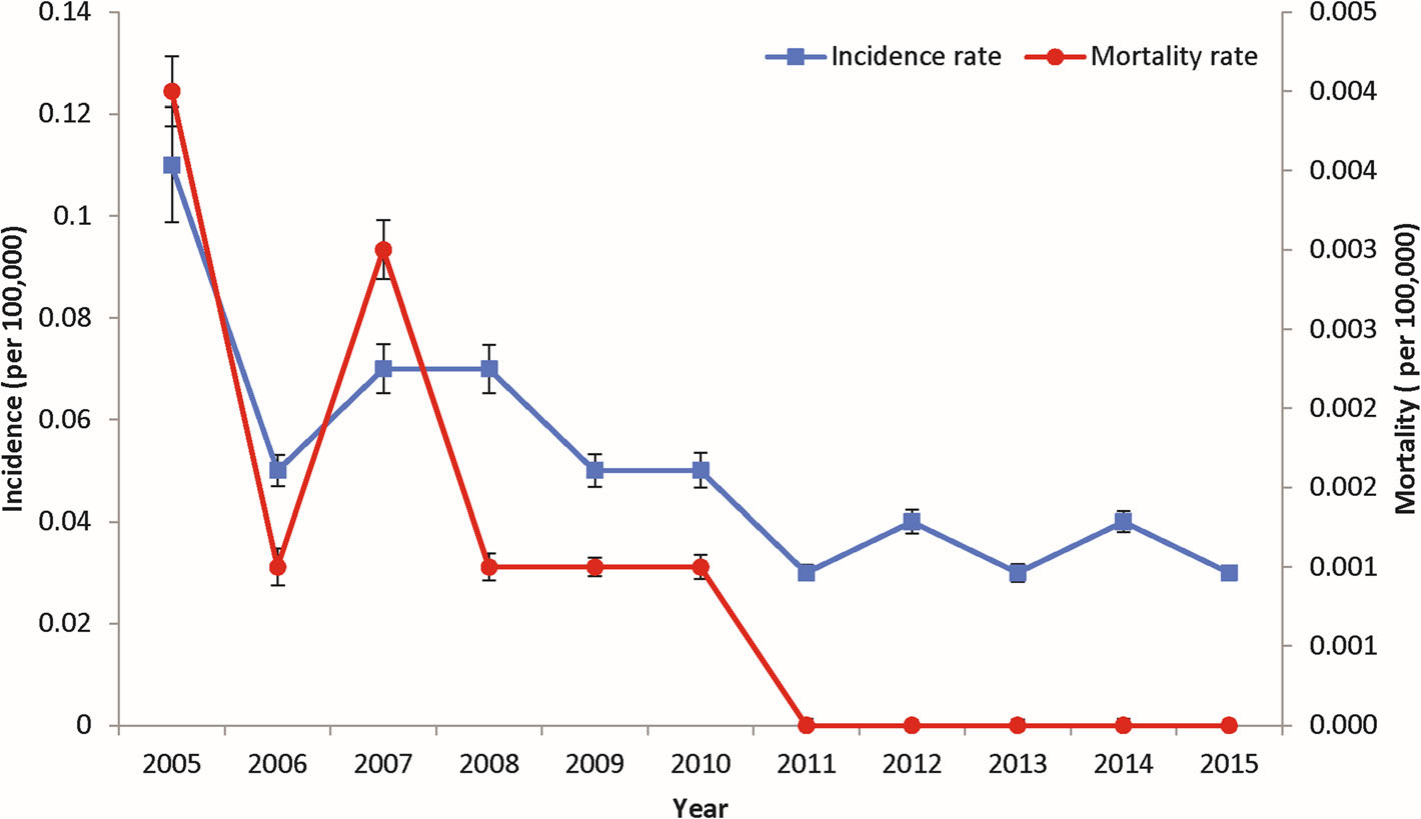
Annual reported incidence and mortality (per 100 000 people) of leptospirosis in China, 2005–2015

The trend in reported incidence and mortality was different between 2005-2010 and 2011–2015 (Fig. 3). A total of 5439 cases were reported in 2005–2010. Subsequently, there were only 2324 cases reported in 2011–2015 (annual IR = 0.03/100 000 people), which was more than 50% lower than the preceding period (*P* < 0.001, Table 3). A slight decreasing trend in reported incidence was identified from 2005 to 2010 (R^2^ = 0.480), while no trend in reported incidence has been observed during 2011–2015 (*P* > 0.05). Statistically, there was a significant difference in reported incidence between age groups during period 2005–2010 (z = 258.51; *P* < 0.001) and period 2011–2015 (z = 50.83; *P* = 0.052). The highest reported incidence rate was observed in the 50–59 age group (0.11/100 000 people). Moreover, there was no significant changes in gender and occupation-specific incidence since 2005 to 2015; the notified incidence remained high in males and primary workforces during both periods (*P* < 0.001).

**Table 3:** Changes in notified incidence and mortality (per 100 000 people) due to leptospirosis in four regions in China during 2005–2010 and 2011–2015

Region	2005–2010				2011–2015			
	No. of cases^a^	Incidence	Deaths	Mortality	No. of cases	Incidence rate	Deaths	Mortality rate
A	835	0.05–0.14	39	0.00–0.01	374	0.04–0.06	4	< 0.01
B	4593	0.00–0.38	107	0.00–0.01	1941	0.00–0.28	18	< 0.01
C	9	< 0.01	0	0	7	< 0.00	0	0
D	2	< 0.00	0	0	2	< 0.00	0	0
Total	5439	0.07	146	0.002	2324	0.03	22	< 0.01

^a^ included all cases (i.e., confirmed, clinical, and suspected)

Of total 168 deaths reported, most deaths were observed (87%, 146/168) during 2005–2010 (Table 3). Few deaths (22 deaths) was recorded during 2011–2015. However, we noted a significant difference in the mortality rate (*P* < 0.001) between 2005 and 2010 and 2011–2015. The mortality rate fluctuated during 2005–2010; where two peaks were observed in 2005 and 2007. In contrast, a relatively stable and low mortality rates was observed during 2011–2015.

Despite the significant drop in notified incidence and number of counties that reported leptospirosis, our analysis indicates 112 new counties were notified leptospirosis infections in 2011–2015 (Additional file 2: Table S5). During this second period, we observed that leptospirosis infection had been reported in new four provinces including Hebei, Inner Mongolia, Jilin, and Gansu (Region C and D).

### Impact of changes in incidence and mortality on the burden of leptospirosis during 2005–2015

It is estimated that during 2005–2015 a total of 10 313 DALYs were lost due to leptospirosis or approximately 937 DALYs per annum (Table 4). Males are the most affected group with an estimated 7149 DALYs or approximately 70% of the total burden. The highest burden estimate was attributed to a group of age 10–19 years, both males and females, accounted for around 30% of the total DALYs. The highest burden estimate was identified in Region B (7990 DALYs), followed by Region A (2312 DALYs) (Table 5).

**Table 4:** Age and gender-specific YLLs, YLDs, and DALYs estimates based on reported leptospirosis in China, 2005–2015

Age	YLLs			YLDs			DALYs		
	Female	Male	Total	Female	Male	Total	Female	Male	Total
0–9	79.31	237.92	317.23	9.50	31.59	41.09	88.81	269.51	358.32
10–19	1077.14	1796.61	2873.75	40.37	163.4	203.77	1117.51	1960.01	3077.52
20–29	566.89	1281.47	1848.36	62.94	151.28	214.22	629.83	1432.75	2062.58
30–39	256.71	824.46	1081.17	117.32	213.98	331.31	374.03	1038.44	1412.48
40–49	255.03	620.29	875.32	132.52	240.34	372.87	387.55	860.63	1248.19
50–59	277.2	695.33	972.53	119.70	244.38	364.08	396.9	939.71	1336.61
60–69	73.73	397.18	470.91	58.90	150.57	209.47	132.63	547.75	680.38
70–79	17.28	52.81	70.09	16.15	42.99	59.14	33.43	95.8	129.23
80–89	0	0	0	2.37	4.75	7.12	2.37	4.75	7.12
90+	0	0	0	0.47	0.24	0.71	0.47	0.24	0.71
TOTAL	2603.29	5906.07	8509.36	560.25	1243.52	1803.77	3163.54	7149.59	10 313.13

**Table 5:** Temporal and geographical distribution of YLLs, YLDs, and DALYs of leptospirosis in China, 2005–2015

	Years of life lost (YLLs)	Years lived with disability (YLDs)	Disability-adjusted life years (DALYs)	DALYs/100 000 people
Year				
2005	2631.51	334.39	2965.90	0.22
2006	1066.17	163.63	1229.80	0.09
2007	1991.61	220.16	2211.77	0.16
2008	813.55	216.36	1029.91	0.08
2009	530.56	153.66	684.22	0.05
2010	345.99	168.38	514.37	0.04
2011	249.07	99.75	348.82	0.03
2012	303.1	116.14	419.24	0.03
2013	245.77	100.94	346.71	0.03
2014	287.34	130.15	417.49	0.03
2015	44.69	100.22	144.91	0.01
Region				
A	2035.89	276.92	2312.81	1.44
B	6473.47	1517.36	7990.83	1.11
C	0	8.55	8.55	< 0.01
D	0	0.95	0.95	< 0.01

Our results indicate a 95% decline in DALYs due to leptospirosis from 2005 to 2015 (*P* < 0.001, Table 5). The highest burden was estimated in 2005 (2966 DALYs), including 2632 YLLs and 334 YLDs; whereas the lowest burden estimates were identified during 2015 (144 DALYs). During 2005–2010, the total burden of leptospirosis was estimated at approximately 8636 DALYs (1439 DALYs per annum). This consisted of 7379 YLLs and 1257 YLDs. It was much higher than the period of 2011–2015, which accounted for approximately 1600 DALYs or a decrease of 80% from the previous period (Additional file 2: Table S6).

**Fig. 4:**
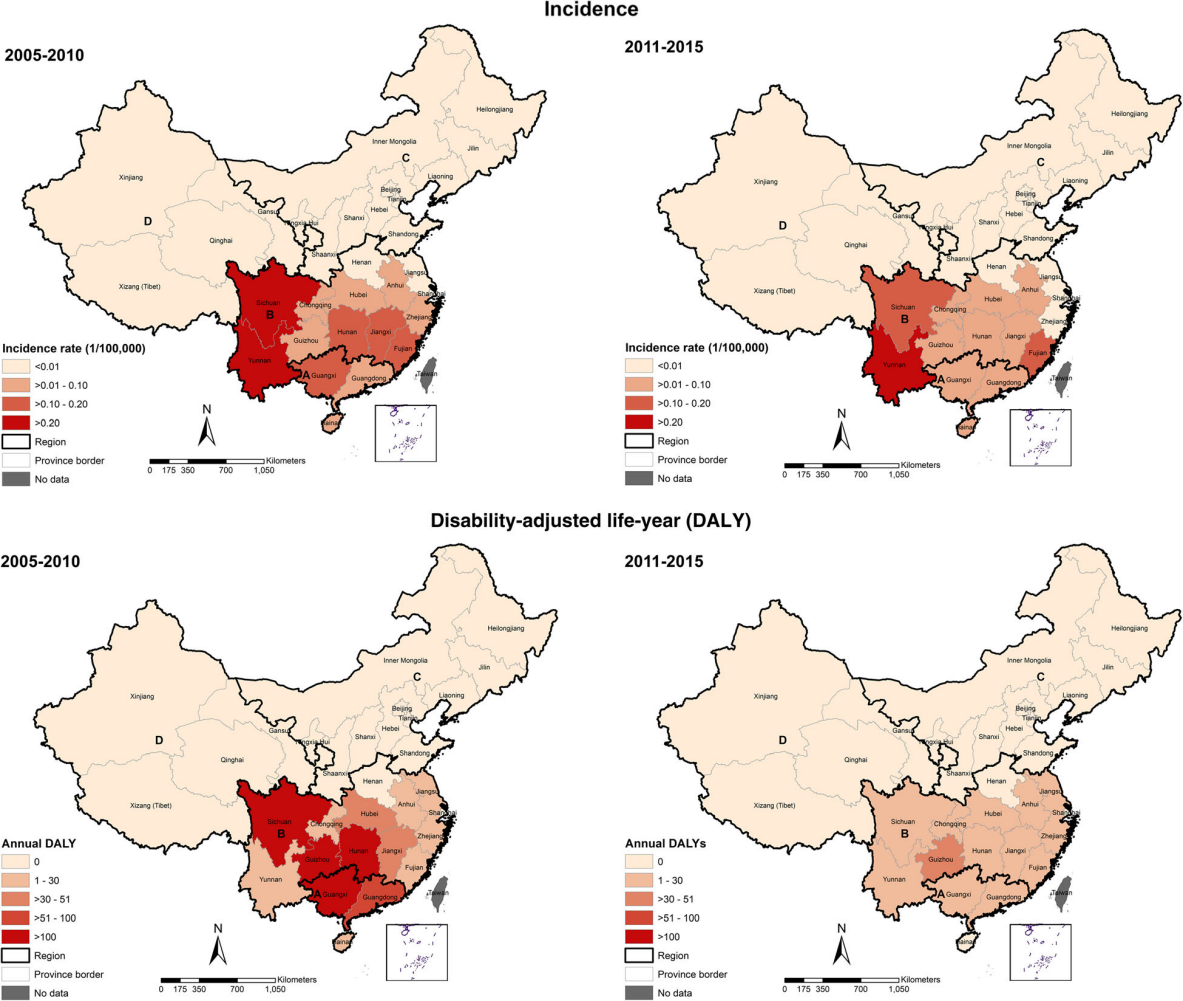
Changes on notified incidence (top) and geographical distribution of the burden (bottom) of leptospirosis in China over two periods, 2005–2010 and 2011–2015

**Fig. 5:**
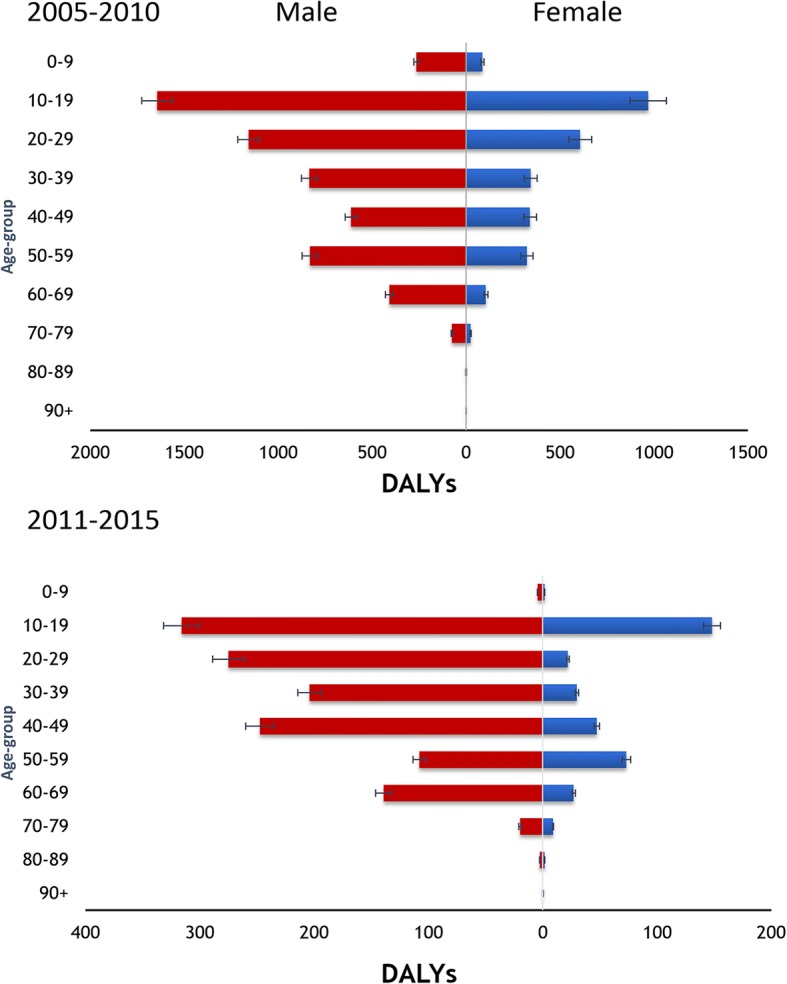
Temporal distribution of the burden estimates of leptospirosis by gender and age groups in China during 2005–2010 and 2011–2015

Between 2005 and 2010 and 2011–2015, a decline in DALY estimate was observed in almost all provinces (Fig. 4). In 2005–2010, high DALYs estimates were observed in Sichuan (1337 DALYs), Guizhou (1936 DALYs), Hunan (1374 DALYs), and Guangxi (1293 DALYs). These four provinces had contributed to approximately 70% of the total DALYs of that period. However, a substantial reduction (on average at 53%) in DALYs occurred in many areas, including in those four provinces during 2011–2015 (*P* < 0.05). Although there was a significant reduction in DALYs, we identify that higher estimates of the burden remain observed in young individuals at the age of 10–19, both sexes (*P* < 0.05, Fig. 5). The burden estimates remained high among males (1316.8 DALYs) compared to females (360.3 DALYs) during 2011–2015 (*P* < 0.05).

A larger quantity of annual YLLs estimates were lost during 2005–2010 (1229 YLLs) compared to the 2011–2015 period (225 YLLs) (*P* < 0.05). Leptospirosis resulted in approximately 5689 YLLs during 2005–2007, and it has contributed 55% of the total DALYs. A three-fold reduction in the number of YLLs was observed in 2005–2006, but a slight increase observed in 2007. Moreover, we found higher YLLs estimates were contributed by economically less-developed provinces such as Sichuan, Guizhou, Hunan, and Guangxi. The highest YLLs estimate was attributed to the younger individual age of 10–19 years. Similarly, YLDs estimates declined over time (*P* < 0.001) and a significant difference on annual YLDs was observed between the two periods (*P* < 0.001). An extended analysis was provided (see Additional file 2: Table S7–Table S10; Additional file 3).

**Fig. 6:**
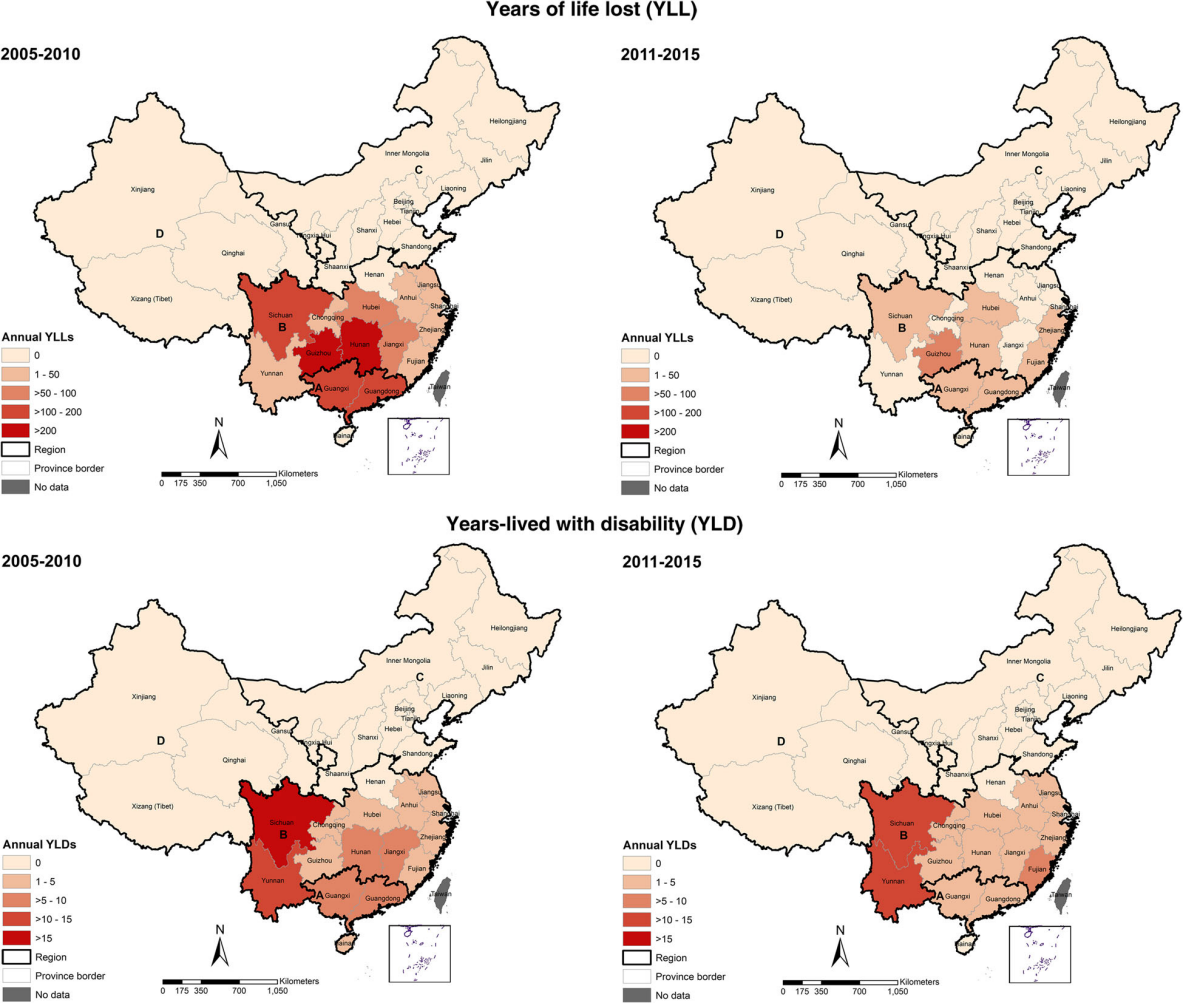
Changes in geographical distribution of years of life lost (YLL) and years-lived with disability (YLD) due to leptospirosis in China during 2005–2015

In terms of YLLs, Guizhou had the highest estimates on YLLs which account for 2300 YLLs or 27% of the total YLLs during 2005–2015, followed by Hunan, Guangxi, and Sichuan (Fig. 6). In Guizhou, the highest YLLs estimates were observed during 2005–2010 (1800 YLLs). During 2011–2015, a dramatic change in YLLs estimates was observed in most provinces. However, the YLLs consistently remained relatively high in Guizhou. In terms of YLDs, Sichuan and Yunnan have higher estimates during both periods.

## Discussion

Our study quantified the remarkable decrease in leptospirosis incidence and mortality in China during 2005–2015, which was accompanied by substantial changes in the demographic and geographic pattern in disease burden estimates. We observed a remarkable decline in reported incidence and mortality in all provinces especially in region A and B where leptospirosis is principally most prevalent, such as Sichuan and Yunnan. These findings are in line with previous county-level studies which also reported a reduction in notified incidence while local outbreaks still frequently reported in some areas [21–26].

Our analysis indicates that following a steep downward trend during 2005–2010, the reported incidence of leptospirosis in China remained quite low during the last five years. This finding suggests that China might have reached a low-level leptospirosis transmission similar to other developed countries [27] although this might not indicate a real epidemiological situation as evidenced by the existence of persistent geographical foci of infection that can potentially lead to future outbreaks in the country. Also, we observed marked variation in fatality-rates across regions over time, which may be explained by the heterogeneity the level of awareness and knowledge among populations towards leptospirosis, inadequate measures for early diagnosis and treatment especially during outbreaks, delay in seeking treatment, and severity of illness due to variation to *Leptospira* exposure and localized risk factors. Hence, more targeted control efforts are needed especially in those high-risk areas and economically less developed areas by enhancing awareness, improving access to safe water and sanitation facilities, and strengthening healthcare and local surveillance systems.

Our study demonstrates an updated burden estimates in terms of DALYs for leptospirosis in China. To the best of our knowledge, this is the first study that attempted to quantify the spatiotemporal heterogeneity in the burden of leptospirosis using time-series historical notification data, especially in China. From 2005 to 2015, it was estimated that more than 10 000 DALYs lost due to infections where the burden predominantly contributed by high YLLs. However, our estimates are in stark contrast with those reported by a study elsewhere [3]. Our smaller DALYs estimates may reflect the sharp reduction in both reported incidence and mortality that occurred during 2005 to 2015.

The burden estimates provided by Torgerson et al. [3] was mainly generated based on the morbidity and mortality estimates developed by another study elsewhere [2] that involved modelling on the morbidity and mortality data by incorporating several variables, adjustments, and uncertainties. Their study was a global study and applied a global model to each country. It is important to consider that epidemiological conditions for leptospirosis transmission and notifications are geographically non-stationary and this approach might be inadequate to capture small-scale heterogeneities. Importantly, that study used published data from 1970s, a period when leptospirosis was highly endemic and when China’s surveillance systems might be different compared to the period of the 2000s. This study extends Torgersen’s [3] study in that we used high-resolution, contemporaneous data based on recent national Chinese disease surveillance systems that have relatively good coverage across the country. This study was successfully demonstrated spatiotemporal heterogeneity in burden within China that was not captured by previous studies. We also identified variation in the surveillance system capacity, as evidenced by the difference in laboratory-confirmed cases among provinces, which means that adjustments should be cautiously applied for the whole country. Applying adjustments to the whole country could be over/underestimated the actual incidence and mortality rates.

Changes in incidence and burden can be partly associated with improvement in prevention and control measures including health promotion activities, sanitation, and the application of leptospirosis vaccination programme [10, 16, 26, 28, 29]. In terms of the surveillance system, the development of NIDRIS and CIDARS following outbreaks of the severe acute respiratory syndrome (SARS) in 2003 have helped efficiently improve the timeliness, completeness, and coverage of the data across China as well as facilitates early detection of diseases outbreaks [16]. However, it has been confirmed that during 2005–2015, there has been no significant change in the surveillance systems, specifically for leptospirosis. While, in terms of the vaccination programme, human leptospirosis vaccine has been developed since 1958 and until now it has been administered to high-risk populations in China during the epidemic seasons. A multivalent inactivated vaccine is currently the only available in China [29].

Changes in ecological and social conditions that have been underway in China in the past 20 years may also have played an essential role in leptospirosis epidemiology. Changes in the landscape, agricultural practices and livestock husbandry, for instance, restriction on livestock herding, farming commercialization, and pigs or livestock vaccination [10, 30] that happened in China could have impacted the transmission rate of leptospirosis in China. Industrialization, for example, has led to significant epidemiological shifts in rural areas through the introduction of agricultural technology and mechanization which might reduce the rate of human exposure to the *Leptospira*-contaminated environment. Also, we had noticed that there were significant anthropogenic ecological changes following the development of the Three Gorges Dam and the nationwide reforestation programme called “Grain for Green”, which probably had an effect of leptospirosis transmission. Water impoundment in many endemic areas has been known to have an impact on rodents’ habitat and population dynamics of the pathogen in those regions [31, 32]. These projects have also been reported to have had a substantial impact on another rodent- and water-borne diseases, such as hemorrhagic fever with renal syndrome and schistosomiasis [33–35]. However, the role of environmental changes on space-time variation on leptospirosis incidence and burden still needs to be explored. Also, a substantial change in the quality of livestock husbandry in China (e.g., improved waste management and farm biosecurity) might also have contributed to a decrease in transmission rate in livestock [10]. A review of significant changes in the epidemiology of infectious diseases in China, including leptospirosis, has also been described elsewhere [14, 36].

Our results demonstrate that the highest DALY was attributed to younger individuals aged 10–19 years due to higher mortality (YLLs) observed in this group. Interestingly, higher DALYs estimates were identified in economically less-developed provinces in China including Guangxi in Region A and Guizhou and Sichuan in Region B. Guangxi and Guizhou are known to have low Gross Regional Product among provinces in China [18]. High burden estimates in school-aged children may probably be associated with lack of parental supervision because of parental migration from rural to the cities that happened during the last three decades. Lack of parental supervision on preventing their children from unhealthy behaviors and environment may likely increase the risk of pathogenic exposure and children’s health [37]. Additionally, rural migration has shifted labor allocation and participation in families in farming activities, where children, women, and elderly become more active in farming [38, 39] and, therefore, they are more likely to be exposed to *Leptospira*-contaminated environments. It has been indicated in our findings that 5% of the total cases among farmers were attributed to young farmers (aged under 20 years). Health education and awareness amongst this population group, especially in rural communities is, therefore essential to further reduce the risk in this demographic group. These findings highlight the importance of improving current local surveillance for leptospirosis and health care services for the high-risk populations living in the high-risk areas identified in our study.

We also found that disease transmission might have emerged in some counties located in temperate regions to the north of the country although at the very low rate. This may partly be due to change in environmental conditions (i.e., climate variation and changes in land use/land cover) or the translocation of potential reservoirs from adjoining endemic regions. Climate variation notably the increase in temperatures may have driven the spread of *Leptospira* towards temperate regions in China through its impact on rodent population growth in these areas [40]. Several outbreaks in China have been thought to be associated with high rainfall intensity [26]. However, we suggest that further investigation should be performed to determine whether the emerging incidence may correlate with changes in climate and other environmental and social conditions (e.g., human migration).

However, our study has several limitations that need to be considered; which mainly associated with the data. First, as there was no information regarding with serovars in our data, we could not be able to analyze further whether the observed change in incidence, mortality and burden was also reflected a dynamic change in circulating serovars in the country during the period of study.

Second, it should also be noted that our burden estimate was based on passive surveillance and all cases reported during the period, which might not represent the actual incidence and burden of disease. The number of reported cases, the proportion of laboratory-confirmed cases, and fatality-rates have shown markedly varied during the period of study and across provinces indicating a variation in diagnostic techniques capacity across the province in the country and therefore could bias our analysis. It should be noted that the surveillance system in China is mainly a hospital-based, but their laboratory capacity to undertake diagnosis through MAT, ELISA, or PCR is also vary across hospitals over the country. Also, since leptospirosis often presenting a broad spectrum of clinical manifestations, more untreated cases or false-negative cases might occur and lead to a high number of underreported and misdiagnosed cases, especially in resource-limited endemic areas. The recent finding indicated the presence of leptospiral infection among patients with undifferentiated fever in Hainan province [41], suggesting that the incidence and burden may be underestimated. Thus, enhanced-surveillance prospective population-based studies may help to determine existing leptospirosis cases in China better.

Another drawback is that there was no detail data available on patient’s clinical presentations in our dataset that we used, so that it was not possible to determine the severity of disease that may help to assess disability weight for DALY estimation as well as to explain the variation of fatality-rates across China. In addition, in this study, we used all categories of cases including suspect, clinical, and confirmed leptospirosis cases as defined by China health authority. It is necessary to acknowledge that there may also be a reporting bias that affects our analysis as leptospirosis have overlapping clinical presentations with another disease (e.g., dengue) [5].

Lastly, the observed reduction in reported incidence and mortality from 2005 to 2015 may also a result of the changes in social and environmental conditions in endemic areas as have been discussed above. In a subsequent study we, therefore, will aim to understand the drivers of such reduction by focusing on high-risk areas and quantify the attributable fraction of determinants such as socioeconomic development, farming practices, and environmental changes.

## Conclusions

In the last 11-years, the burden estimates of leptospirosis indicated a declining trend across the country; however, leptospirosis should not be neglected as it remains an important zoonotic disease and potentially disproportionately affected the young and productive population group in China. In addition, while in the last five years the incidence has been reported at very low-level, this might not reflect the actual incidence of leptospirosis. Active surveillance studies on acute febrile illnesses are urgently required. Finally, these findings may help to design targeted intervention strategies in China to reduce the burden of human leptospirosis. An evaluation of the role of environment and socioeconomic on the changing leptospirosis epidemiology and burden should be considered in the future works.

## Additional files

Additional file 1: Multilingual abstract in the five official working languages of the United Nations. (PDF 778 kb)

Additional file 2: **Table S1.** Temporal distribution of reported leptospirosis incidence in four regions in China by province, 2005–2015. **Table S2.** Reported leptospirosis cases and the proportion of laboratory-confirmed case (in percent) by type of occupational group, China, 2005–2015. **Table S3.** Case fatality-rates (CFR) of leptospirosis by province in two regions in China, 2005–2015. **Table S4.** Temporal distribution of notified mortality rates due to leptospirosis in two regions in China, by province, 2005–2015. **Table S5.** Number of counties reported leptospirosis each year and new counties that reported leptospirosis during 2005–2010 and 2011–2015. **Table S6.** Disability-adjusted life-years (DALYs) estimates of leptospirosis by gender, by age and year, China. **Table S7.** Temporal trend of years of life lost (YLL) due to leptospirosis in China, by gender, by age and year. **Table S8.** Years of life lost (YLLs) estimates for leptospirosis in China, by region, gender, by age period of year. **Table S9.** Years-lived with disability (YLD) due to leptospirosis in China, by gender, by age and year. **Table S10.** Geographical distribution of years of life lost (YLL), years-lived with disability (YLD), and disability-adjusted life years (DALY) by region during both periods in China. (DOCX 64 kb)

Additional file 3: The distribution of age-, sex-, and geographically-specific years of life lost (YLLs) estimates in China, 2005–2015. (XLSX 23 kb)

## Abbreviations

CDC: Center for Disease Control and Prevention
CFR: Case fatality-rate
CIDARS: China Infectious Disease Automated-alert and Response System
CISDCP: China Information System for Disease Control and Prevention
DALY: Disability-adjusted life-year
DW: Disability weight
ELISA: Enzyme-linked immunosorbent assay
IR: Incidence rate
MAT: Microscopic agglutination test
MR: Mortality rate
NHFPC: National Health and Family Planning Commission
NIDRIS: Notifiable Infectious Diseases Reporting Information System
PCR: Polymerase chain reaction
SARS: Severe acute respiratory syndrome
WHO: World Health Organization
YLD: Years-lived with disability
YLL: Years of life-lost
