# Supplementary material for: Epidemiological shift and geographical heterogeneity in the burden of leptospirosis in China
Source: Infect Dis Poverty. 2018 May 18;7:57. doi: 10.1186/s40249-018-0435-2 (PMC5985562; doi:10.1186/s40249-018-0435-2)
Supplement: Supplementary file 1 — Multilingual abstract in the five official working languages of the United Nations. (PDF 778 kb) [file 40249_2018_435_MOESM1_ESM.pdf]

Translation of the abstract into the five official working languages of the United Nations

الاتجاهات في علم الأوبئة لداء البريميات في الصين وأثره على التقديرات السنوية لأعباء الأمراض

قدمه: بانجي ويباوا ديوانتارا، وعبد الله المأمون، ووين-يي تشانغ، ووين-وو ين، وفان دينغ، ودان-هاييجو، ووين-بي ها، وفيدريكو كوستا، وألبرت إيكسانج كو، وريكاردو جي سواريس ماجالهايس

الملخص

المعلومات الأساسية: انخفضت معدلات المرض والوفيات بداء البريميات في الصين منذ العقد الأول من القرن الحادي والعشرين. هناك حاجة إلى مزيد من التحليلات للتغيرات الزمانية المكانية والديموغرافية التي حدثت في العقد الماضي وآثارها على تقديرات عبء المرض لإبلاغ استراتيجيات التدخل. في هذه الدراسة ، قمنا بتحديد مقدار التحول الوبائي والتجانس الجغرافي في عبء داء البريميات أثناء الفترة 2005-2015 في الصين.

الطرق:استخدمنا بيانات حالة داء البريميات في الفترة من 1 يناير 2005 إلى 31 ديسمبر 2015 والتي تم جمعها بشكل روتيني من قبل نظام معلومات الصين لمكافحة الأمراض والوقاية منها (CISDCP) لتحليل الاتجاه الوبائي وتقدير العبء من حيث سنوات العمر المعدلة حسب الإعاقة (DALYs) على المكان والوقت والمجموعات السكانية.

النتائج: تم الإبلاغ عن 7763 حالة خلال الفترة 2005-2015. منها 2403 حالة (31٪) هي الحالات المؤكدة مختبريًا. منذ عام 2005 ، انخفض معدل حدوث الإخطار تدريجيا (القيمة الاحتمالية < 0.05) وكان مستقرًا نسبيًا خلال الفترة 2011-2015 (القيمة الاحتمالية > 0.05). خلال الفترة 2005 - 2015 ، قدرنا أن إجمالي 10313 من سنوات العمر المعدلة حسب الإعاقة تم احتسابها مدد ضاعت بسبب داء البريميات والتي تضم مجموع 1804 سنة حياة مع العجز (YLDs) و 8509 سنة من الحياة مفقودة (YLLs). كان للذكور أعلى عبء للمرض (DALY 7149) مقارنة بالإناث (DALYs 3164). ويُعزى أعلى تقدير للأعباء إلى الأفراد الأصغر سنا الذين تتراوح أعمارهم بين 10 و 19 سنة والذين يعيشون في المقاطعات الجنوبية من الصين. خلال الفترة 2005-2015 ، ساهمت هذه الفئة العمرية في حوالي 3078 سنة من سنوات العمر المعدلة حسب الإعاقة بما يعادل 30٪ من إجمالي فقدان سنوات العمر المعدلة حسب الإعاقة في الصين. ومع ذلك ، أشار تحليلنا إلى وجود اتجاه تنازلي في تقديرات العبء (القيمة الاحتمالية < 0.001) منذ عام 2005 وبقيت منخفضة نسبيًا خلال الفترة 2011-2015. وقد تم تحديد تقديرات عبء منخفض في المناطق الموبوءة حيث تنتشر العدوى في الأساس. كانت معظم التغييرات في تقديرات سنوات العمر المعدلة حسب الإعاقة DALY مدفوعة بالتغييرات في سنوات العمر المفقودة YLLs.

الاستنتاجات: في السنوات ال 11 الماضية ، أظهرت تقديرات عبء داء البريميات انخفاض الاتجاه في جميع أنحاء البلاد. ومع ذلك ، لا ينبغي إهمال داء البريميات لأنه لا يزال مرضًا حيوانيًا هامًا ، ويمكن أن يؤثر على السكان الشباب والمنتجين في المقاطعات الأقل نموًا اقتصاديًا في جنوب الصين. بالإضافة إلى ذلك ، في حين أنه في السنوات الخمس الأخيرة تم الإبلاغ عن حدوث المرض بمستوى منخفض للغاية ، فإن ذلك قد لا يعكس الوقوع الحقيقي لداء البريميات. ومن ثم ، فإن تعزيز المراقبة في المناطق الموبوءة هو المطلوب إلى حد كبير للسيطرة على الانتشار الفعلي من أجل مكافحة داء البريميات في الصين بشكل أفضل.

Translated from English version into Arabic by Free bird, proofread by Bashaier Allam, through

TRANSLATORS
WITHOUT BORDERS

中国钩端螺旋体病负担的流行病学变化及地理异质性

Pandji Wibawa Dhewantara, Abdullah Al Mamun, Wen-Yi Zhang, Wen-Wu Yin, Fan Ding, Danhuai Guo, Wenbiao Hu, Federico Costa, Albert Icksang Ko, Ricardo J. Soares Magalhães

摘要
引言：自 2000 年以来，中国的钩端螺旋体病发病率和死亡率有所下降。分析近十年间钩端螺旋体病时空、人口特征发生的变化及其对评估疾病负担的意义，为干预策略提供依据。
方法：我们使用 2005 年 1 月 1 日至 2015 年 12 月 31 日期间中国疾病预防控制中心传染病报告信息管理系统常规报告的钩端螺旋体病病例资料来分析不同时间、不同空间和不同人群的流行病学趋势，并评估伤残调整寿命年的负担。
结果：2005 至 2015 年全国累计报告钩端螺旋体病病例 7763 例，其中 2403 例（31%）为实验室确诊病例。自 2005 年以来钩端螺旋体病报告发病率呈逐渐下降趋势（$P < 0.05$），2011-2015 年间发病率相对稳定（$P > 0.05$）。2005-2015 年期间，由钩端螺旋体病引起的伤残调整寿命年共 10313 人年，其中包括伤残损失寿命年为 1804 人年，减寿年数为 8509 人年。男性病例伤残调整寿命年总数高于女性。中国南部省份的 10-19 岁年轻人疾病负担最高，且伤残调整寿命年共约 3078 人年，占中国伤残调整寿命年总数的 30%。分析显示自 2005 年以来疾病负担呈现下降趋势，并在 2011-2015 年保持相对较低的水平。在感染主要分布的流行地区，本研究确定了较低的疾病负担估计。估计大部分伤残调整寿命年变化均由减寿年数的变化引起。
结论：近 11 年中全国钩端螺旋体病的负担估计呈下降趋势，但它仍然是一种重要的人畜共患疾病，潜在影响中国南部经济欠发达省份年轻劳动力人口的健康，因此钩端螺旋体病不容忽视。此外，虽然在过去五年中报告发病率保持较低水平，但这并不能反映钩端螺旋体病的实际发病率。因此，为了更好地控制中国的钩端螺旋体病必须在流行地区加强监测以获取实际的患病率。

Translated from English version into Chinese by Wen-Yi Zhang and Fan Ding

Tendances de l'épidémiologie de la leptospirose en Chine et impact sur les estimations annuelles du fardeau de la maladie

Pandji Wibawa Dhewantara, Abdullah Al Mamun, Wen-Yi Zhang, Wen-Wu Yin, Fan Ding, Dan-Huai Guo, Wen-Biao Hu, Federico Costa, Albert Icksang Ko et Ricardo J Soares Magalhães

Résumé
Contexte: Les taux de morbidité et de mortalité liés à la leptospirose sont en diminution en Chine depuis les années 2000. De nouvelles analyses des changements spatiotemporels et démographiques survenus au cours de la dernière décennie et de leurs implications sur les estimations du fardeau de la maladie sont nécessaires pour informer les stratégies d'intervention. Dans la présente étude, nous avons quantifié le changement épidémiologique et l'hétérogénéité géographique du fardeau de la leptospirose en Chine entre 2005 et 2015.
Méthodes: Nous avons utilisé les données des cas de leptospirose rapportés entre le 1er janvier 2005 et le 31 décembre 2015, qui ont été systématiquement collectées par le Système d'information chinois pour le contrôle et la prévention des maladies (CISDCP), afin d'analyser les tendances épidémiologiques et d'estimer le fardeau de la maladie en termes d'années d'espérance de vie corrigée par le handicap (DALY) aux échelles spatiale, temporelle et des groupes démographiques.
Résultats: Au total, 7763 cas ont été rapportés entre 2005 et 2015. Parmi ceux-ci, 2403 cas (31%) ont été confirmés par les analyses en laboratoire. Le taux d'incidence rapporté a progressivement diminué à

partir de 2005 (*P* < 0,05), avant de rester relativement stable entre 2011 et 2015 (*P* > 0,05). Entre 2005 et 2015, on estime que 10313 DALY ont été perdues à cause de la leptospirose, soit 1804 années de vie avec un handicap (YLD) et 8509 années de vie perdues (YLL). Les hommes supportent un plus fort fardeau de la maladie (7149 DALY) que les femmes (3164 DALY). Le plus fort fardeau estimé pèse sur les jeunes sujets âgés de 10 à 19 ans et vivant dans les provinces du sud de la Chine. Entre 2005 et 2015, cette tranche d'âge représente environ 3078 DALY, soit environ 30% du total des DALY perdues en Chine. Cependant, notre analyse indique une tendance à la diminution du fardeau estimé (*P* < 0,001) à partir de 2005 et son maintien à des valeurs relativement basses entre 2011 et 2015. Des estimations basses du fardeau de la maladie ont été relevées dans les régions d'endémie, où se produisent la majorité des infections. La plupart des changements dans les estimations de DALY résultent de changements des années de vie perdues.

Conclusions: Au cours des 11 dernières années, le fardeau estimé de la leptospirose tend à diminuer dans toute la Chine. Il ne faut pas pour autant la négliger car elle reste une maladie zoonotique importante, susceptible d'affecter les populations jeunes et productives des provinces du sud, moins développées économiquement. De plus même, si l'incidence rapportée est restée faible au cours des cinq dernières années, cela peut ne pas refléter la véritable incidence de la leptospirose. Une surveillance renforcée dans les régions d'endémie est par conséquent très nécessaire afin d'avoir une idée de la véritable prévalence et de mieux lutter contre la leptospirose en Chine.

Translated from English version into French by Suzanne Assenat, proofread by Charlote Giovan, through

TRANSLATORS
WITHOUT BORDERS

Тенденции в эпидемиологии лептоспироза в Китае и его влияния на оценку ежегодного бремени болезней

Панджи Вибава Дхевантара, Абдуллах аль-Мамун, Вэнь-И Чжан, Вэнь-У Инь, Фань Дин, Дань-Хуай Го, Вэнь-Бяо Ху, Федерико Коста, Альберт Иксанг Ко и Рикарду Ж. Суареш Магальяйнш

Аннотация

Справочная информация: С 2000-х годов в Китае наблюдается снижение уровня заболеваемости и смертности, связанных с лептоспирозом. Для обоснования стратегий вмешательства требуется более глубокое изучение как произошедших за последнее десятилетие пространственно-временных и демографических изменений, так и их влияния на оценку бремени заболевания. Данное исследование посвящено количественному выражению эпидемиологических сдвигов и географической гетерогенности в бремени лептоспироза за период с 2005 по 2015 гг. в Китае.

Методы: В работе использовались данные о зарегистрированных случаях лептоспироза за период с 1го января 2005 года по 31е декабря 2015 года, регулярно получаемые из

Информационной системы по контролю и профилактике заболеваний Китая (CISDCP), с целью изучения эпидемиологических тенденций, а также для оценки бремени с учётом лет жизни с поправкой на инвалидность (DALYs) по месту, времени и демографическим группам.

Результаты: За период с 2005 по 2015 годы было зарегистрировано всего 7763 случаев заболевания. Из которых 2403 (31%) случая были подтверждены в лабораторных условиях. С 2005 года объявленный уровень заболеваемости постепенно снижался (P < 0,05) и оставался относительно стабильным в период с 2011 по 2015 гг. (P > 0,05). Было определено, что в период с 2005 по 2015 гг. вследствие лептоспироза было утеряно в общей сложности 10 313 DALYs, что составило в целом 1804 лет жизни в состоянии инвалидности (YLDs) и 8509 утраченных лет жизни (YLLs). Наивысшее бремя болезни приходилось на мужчин (7149 DALYs) по сравнению с женщинами (3164 DALYs). Наивысшее значение показателя бремени наблюдалось среди молодых людей в возрасте от 10 до 19 лет, проживавших в южных провинциях Китая. В период с 2005 по 2015 гг указанная возрастная группа составила приблизительно 3078 DALYs, что соответствует 30% общей потери DALYs в Китае. Тем не менее, настоящий анализ показал тенденцию снижения показателя бремени (P < 0,001) с 2005 года и относительную стабильность на низком уровне в период с 2011 по 2015 гг. Низкое значение показателя бремени было выявлено в эндемичных регионах, где главным образом распределялось заражение. Большинство изменений в оценке DALY были вызваны изменениями в YLLs.

Заключение: Оценка бремени лептоспироза за последние 11 лет продемонстрировала тенденцию снижения заболевания по всей стране, тем не менее, данное заболевание не следует игнорировать, поскольку оно остаётся важной зоонозной болезнью и способно влиять на молодое и трудоспособное население в менее развитых в экономическом плане провинциях на юге Китая. Вдобавок, несмотря на то, что за последние пять лет зарегистрированная заболеваемость находилась на низком уровне, это может не соответствовать реальному уровню заболеваемости лептоспирозом. Следовательно, требуется существенное усиление надзора в эндемичных регионах с целью выявления фактической заболеваемости, чтобы улучшить контроль за лептоспирозом в Китае.

Translated from English version into Russian by Liudmila Tomanek, proofread by Sam Abramov, through

TRANSLATORS
WITHOUT BORDERS

Tendencias en la epidemiología de la leptospirosis en China y su impacto en la estimación anual de la carga de enfermedades

Pandji Wibawa Dhewantara, Abdullah Al Mamun, Wen-Yi Zhang, Wen-Wu Yin, Fan Ding, Dan-Huai Guo, Wen-Biao Hu, Federico Costa, Albert Icksang Ko y Ricardo J Soares Magalhães

Resumen

Antecedentes: Las tasas de morbilidad y mortalidad por leptospirosis han disminuido en China desde la década del 2000. Se requieren estudios adicionales de cambios espacio-temporales y demográficos que hayan tenido lugar en la última década, así como su implicación en la estimación de la carga de las enfermedades para informar sobre estrategias de intervención. En este estudio, se han cuantificado el cambio epidemiológico y la heterogeneidad geográfica en lo que respecta a la carga de la leptospirosis en China entre los años 2005 y 2015.

Métodos: Se utilizaron datos de casos reportados de leptospirosis desde el día 1 de enero de 2005 hasta el 31 de diciembre de 2015 que fueron recolectados de forma rutinaria por el Sistema de información chino para el control y prevención de enfermedades (CISDCP, por sus siglas en inglés), para analizar la tendencia epidemiológica y estimar la carga en materia de años de vida ajustados en función de la discapacidad (AVAD) sobre el espacio, el tiempo y los grupos demográficos.

Resultados: Se reportaron un total de 7763 casos entre los años 2005 y 2015. De los que 2403 (31%) eran casos confirmados de laboratorio. Desde el año 2005, la tasa de notificación de incidencias ha disminuido ($P < 0.05$) y fue relativamente estable entre los años 2011 y 2015 ($P > 0.05$). Entre los años 2005 y 2015, se estimó una pérdida total de 10 313 AVAD por leptospirosis que incluían un total de 1804 años de vida con discapacidad (AVD) y 8509 años de vida perdidos por muerte prematura (AVP). Los hombres tienen la carga más elevada de enfermedad (7149 AVAD) comparado con las mujeres (3164 AVAD). La carga más alta estimada fue atribuida a individuos jóvenes de entre 10 y 19 años que vivían en provincias del sur de China. Entre los años 2005 y 2015, este grupo de edad contribuyó a aproximadamente 3078 AVAD, que corresponden con el 30% del total de AVAD perdidos en China. Aun así, los análisis indicaban una tendencia a la baja en la estimación de la carga ($P < 0.001$) desde 2005 y permanecieron relativamente bajos entre los años 2011 y 2015. La baja estimación de la carga ha sido identificada en regiones endémicas en las que se propagaban principalmente las infecciones. La mayoría de cambios de estimación en AVAD se efectuaron por cambios en AVP.

Conclusiones: En los últimos 11 años, la estimación de la carga de leptospirosis ha mostrado una tendencia a la baja a lo largo del país. Sin embargo, la leptospirosis no se debería desatender, ya que continúa siendo una enfermedad zoonótica importante y que afecta de forma potencial a la población joven y productiva de provincias económicamente menos desarrolladas en el sur de China. Además, a pesar de que en los últimos años la incidencia ha sido reportada a niveles muy bajos, esto no refleja la verdadera incidencia de la leptospirosis. La vigilancia reforzada en regiones endémicas es, por lo tanto, sustancialmente requerida para captar la prevalencia real para un mejor control de la leptospirosis en China.

Translated from English version into Spanish by Sílvia Calatayud, proofread by Lucía Leunda Tosi, through

TRANSLATORS
WITHOUT BORDERS
