# Supplementary material for: Epidemiological shift and geographical heterogeneity in the burden of leptospirosis in China
Source: Infect Dis Poverty. 2018 May 18;7:57. doi: 10.1186/s40249-018-0435-2 (PMC5985562; doi:10.1186/s40249-018-0435-2)
Supplement: Supplementary file 2 — Table S1. Temporal distribution of reported leptospirosis incidence in four regions in China by province, 2005–2015. Table S2. Reported leptospirosis cases and the proportion of laboratory-confirmed case (in percent) by type of occupational group, China, 2005–2015. Table S3. Case fatality-rates (CFR) of leptospirosis by province in two regions in China, 2005–2015. Table S4. Temporal distribution of notified mortality rates due to leptospirosis in two regions in China, by province, 2005–2015. Table S5. Number of counties reported leptospirosis each year and new counties that reported leptospirosis during 2005–2010 and 2011–2015. Table S6. Disability-adjusted life-years (DALYs) estimates of leptospirosis by gender, by age and year, China. Table S7. Temporal trend of years of life lost (YLL) due to leptospirosis in China, by gender, by age and year. Table S8. Years of life lost (YLLs) estimates for leptospirosis in China, by region, gender, by age period of year. Table S9. Years-lived with disability (YLD) due to leptospirosis in China, by gender, by age and year. Table S10. Geographical distribution of years of life lost (YLL), years-lived with disability (YLD), and disability-adjusted life years (DALY) by region during both periods in China. (DOCX 64 kb) [file 40249_2018_435_MOESM2_ESM.docx]

**Table S1.** **Temporal distribution of reported leptospirosis incidence in four regions in China by province, 2005-2015**

| **Region** | **Province** | **Number of reported cases (Cases per 100 000 population)** | | | | | | | | | | | **Total No of cases (% confirmed)*** | **Annual**  **IR** |
| --- | --- | --- | --- | --- | --- | --- | --- | --- | --- | --- | --- | --- | --- | --- |
|  |  | **2005** | **2006** | **2007** | **2008** | **2009** | **2010** | **2011** | **2012** | **2013** | **2014** | **2015** |  |  |
| **A** | Guangdong | 65 (0.07) | 69 (0.08) | 64 (0.07) | 83 (0.09) | 60 (0.06) | 59 (0.06) | 59 (0.06) | 43 (0.04) | 53 (0.05) | 30 (0.03) | 34 (0.03) | 619 (53) | 0.06 |
|  | Guangxi | 72 (0.15) | 66 (0.14) | 62 (0.13) | 86 (0.18) | 66 (0.14) | 56 (0.12) | 28 (0.06) | 36 (0.08) | 24 (0.05) | 28 (0.06) | 19 (0.04) | 543 (40) | 0.10 |
|  | Hainan | 7 (0.08) | 5 (0.06) | 4 (0.05) | 4 (0.05) | 4 (0.05) | 3 (0.03) | 4 (0.05) | 5 (0.06) | 3 (0.03) | 6 (0.07) | 2 (0.02) | 47 (11) | 0.05 |
|  | *Sub-total* | 144 (0.10) | 140 (0.09) | 130 (0.08) | 173 (0.11) | 130 (0.08) | 118 (0.07) | 91 (0.06) | 84 (0.06) | 80 (0.05) | 64 (0.05) | 55 (0.03) | 1,209 (45) | 0.07 |
|  |  |  |  |  |  |  |  |  |  |  |  |  |  |  |
| **B** | Jiangsu | 13 (0.02) | 6 (0.01) | 7 (0.01) | 5 (0.01) | 1 (0.00) | 1 (0.00) | 4 (0.01) | 0 (0.00) | 0 (0.00) | 0 (0.00) | 0 (0.00) | 37 (54) | 0.00 |
|  | Zhejiang | 14 (0.03) | 8 (0.02) | 53 (0.11) | 9 (0.02) | 6 (0.01) | 17 (0.03) | 8 (0.01) | 2 (0.00) | 6 (0.01) | 7 (0.01) | 8 (0.01) | 138 (43) | 0.02 |
|  | Anhui | 25 (0.04) | 18 (0.03) | 56 (0.09) | 55 (0.09) | 32 (0.05) | 47 (0.08) | 24 (0.04) | 19 (0.03) | 9 (0.01) | 18 (0.03) | 7 (0.01) | 310 (8) | 0.05 |
|  | Fujian | 40 (0.11) | 26 (0.07) | 33 (0.09) | 43 (0.12) | 53 (0.15) | 49 (0.13) | 45 (0.12) | 47 (0.12) | 59 (0.16) | 54 (0.14) | 53 (0.14) | 502 (47) | 0.12 |
|  | Henan | 0 (0.00) | 3 (0.00) | 0 (0.00) | 0 (0.00) | 0 (0.00) | 0 (0.00) | 0 (0.00) | 0 (0.00) | 0 (0.00) | 0 (0.00) | 0 (0.00) | 3 (0) | 0.00 |
|  | Jiangxi | 89 (0.21) | 54 (0.13) | 58 (0.13) | 61 (0.14) | 47 (0.11) | 27 (0.06) | 30 (0.07) | 17 (0.04) | 19 (0.04) | 17 (0.04) | 2 (0.00) | 421 (5) | 0.09 |
|  | Hubei | 38 (0.07) | 22 (0.04) | 54 (0.09) | 97 (0.17) | 17 (0.03) | 19 (0.03) | 15 (0.03) | 9 (0.02) | 6 (0.01) | 6 (0.01) | 13 (0.02) | 296 (11) | 0.05 |
|  | Hunan | 74 (0.12) | 92 (0.15) | 150 (0.24) | 79 (0.12) | 39 (0.06) | 41 (0.06) | 33 (0.05) | 41 (0.06) | 30 (0.05) | 36 (0.05) | 41 (0.06) | 656 (16) | 0.09 |
|  | Chongqing | 56 (0.20) | 13 (0.05) | 20 (0.07) | 28 (0.10) | 24 (0.08) | 13 (0.05) | 8 (0.03) | 10 (0.03) | 7 (0.02) | 6 (0.02) | 15 (0.05) | 200 (10) | 0.06 |
|  | Sichuan | 792 (0.96) | 155 (0.19) | 218 (0.27) | 237 (0.29) | 203 (0.25) | 255 (0.31) | 73 (0.09) | 145 (0.18) | 98 (0.12) | 110 (0.14) | 66 (0.08) | 2352 (6) | 0.26 |
|  | Guizhou | 56 (0.15) | 45 (0.12) | 42 (0.11) | 31 (0.08) | 16 (0.04) | 18 (0.05) | 14 (0.04) | 30 (0.09) | 7 (0.02) | 8 (0.02) | 24 (0.07) | 291 (12) | 0.07 |
|  | Yunnan | 114 (0.26) | 130 (0.29) | 135 (0.30) | 109 (0.24) | 87 (0.19) | 109 (0.24) | 77 (0.17) | 82 (0.18) | 110 (0.24) | 226 (0.48) | 129 (0.27) | 1,308 (86) | 0.26 |
|  | *Sub-total* | 1,311 (0.18) | 572 (0.09) | 826 (0.13) | 754 (0.12) | 525 (0.08) | 596 (0.09) | 331 (0.05) | 402 (0.06) | 351 (0.06) | 488 (0.08) | 358 (0.06) | 6,514 (28) | 0.09 |
|  |  |  |  |  |  |  |  |  |  |  |  |  |  |  |
| **C** | Beijing | 0 (0.00) | 0 (0.00) | 0 (0.00) | 1 (0.01) | 1 (0.01) | 0 (0.00) | 0 (0.00) | 0 (0.00) | 0 (0.00) | 0 (0.00) | 0 (0.00) | 2 (50) | 0.00 |
|  | Shandong | 1 (0.00) | 2 (0.00) | 0 (0.00) | 0 (0.00) | 1 (0.00) | 5 (0.01) | 2 (0.00) | 2 (0.00) | 0 (0.00) | 5 (0.01) | 2 (0.00) | 20 (65) | 0.00 |
|  | Hebei | 0 (0.00) | 0 (0.00) | 0 (0.00) | 0 (0.00) | 0 (0.00) | 0 (0.00) | 1 (0.00) | 0 (0.00) | 0 (0.00) | 1 (0.00) | 1 (0.00) | 3 (33) | 0.00 |
|  | Shanxi | 1 (0.00) | 0 (0.00) | 2 (0.01) | 0 (0.00) | 0 (0.00) | 0 (0.00) | 0 (0.00) | 0 (0.00) | 0 (0.00) | 0 (0.00) | 0 (0.00) | 3 (33) | 0.00 |
|  | Inner Mongolia | 0 (0.00) | 0 (0.00) | 0 (0.00) | 0 (0.00) | 0 (0.00) | 0 (0.00) | 0 (0.00) | 1 (0.00) | 0 (0.00) | 0 (0.00) | 0 (0.00) | 1 (100) | 0.00 |
|  | Liaoning | 1 (0.00) | 0 (0.00) | 0 (0.00) | 0 (0.00) | 0 (0.00) | 0 (0.00) | 0 (0.00) | 0 (0.00) | 0 (0.00) | 0 (0.00) | 0 (0.00) | 1 (0) | 0.00 |
|  | Jilin | 0 (0.00) | 0 (0.00) | 0 (0.00) | 0 (0.00) | 0 (0.00) | 0 (0.00) | 2 (0.01) | 0 (0.00) | 0 (0.00) | 0 (0.00) | 0 (0.00) | 2(100) | 0.00 |
|  | Shaanxi | 0 (0.00) | 0 (0.00) | 1 (0.00) | 0 (0.00) | 2 (0.01) | 0 (0.00) | 0 (0.00) | 0 (0.00) | 1 (0.00) | 0 (0.00) | 0 (0.00) | 4 (25) | 0.00 |
|  | *Sub-total* | 3 (0.00) | 2 (0.00) | 3 (0.00) | 1 (0.00) | 4 (0.00) | 5 (0.00) | 5 (0.00) | 3 (0.00) | 1 (0.00) | 6 (0.00) | 3 (0.00) | 36 (55) | 0.00 |
|  |  |  |  |  |  |  |  |  |  |  |  |  |  |  |
| **D** | Gansu | 0 (0.00) | 0 (0.00) | 0 (0.00) | 0 (0.00) | 0 (0.00) | 0 (0.00) | 0 (0.00) | 0 (0.00) | 0 (0.00) | 1 (0.00) | 0 (0.00) | 1 (100) | 0.00 |
|  | Qinghai | 0 (0.00) | 0 (0.00) | 0 (0.00) | 1 (0.02) | 0 (0.00) | 0 (0.00) | 0 (0.00) | 0 (0.00) | 0 (0.00) | 0 (0.00) | 0 (0.00) | 1 (0) | 0.00 |
|  | Xinjiang | 0 (0.00) | 0 (0.00) | 0 (0.00) | 1 (0.00) | 0 (0.00) | 0 (0.00) | 0 (0.00) | 1 (0.00) | 0 (0.00) | 0 (0.00) | 0 (0.00) | 2 (0) | 0.00 |
|  | *Sub-total* | 0 (0.00) | 0 (0.00) | 0 (0.00) | 2 (0.00) | 0 (0.00) | 0 (0.00) | 0 (0.00) | 1 (0.00) | 0 (0.00) | 1 (0.00) | 0 (0.00) | 4 (25) | 0.00 |
|  |  |  |  |  |  |  |  |  |  |  |  |  |  |  |
| **Morbidity** | | 1,458 (0.11) | 714 (0.05) | 959 (0.07) | 930 (0.07) | 659 (0.05) | 719 (0.05) | 427 (0.03) | 490 (0.04) | 432 (0.03) | 559 (0.04) | 416 (0.03) | 7,763 (31) | 0.05 |

* number of cases; percent of reported laboratory-confirmed cases (in parenthesis)

**Table S2. Reported leptospirosis cases and confirmed case (in percent) by type of occupational group, China, 2005-2015**

| **Occupational group** | | **No. of reported case (% confirmed case)** | | | | | | | | | | | **Total (% confirmed case)** |
| --- | --- | --- | --- | --- | --- | --- | --- | --- | --- | --- | --- | --- | --- |
|  |  | **2005** | **2006** | **2007** | **2008** | **2009** | **2010** | **2011** | **2012** | **2013** | **2014** | **2015** |  |
| Primary sector (Agriculture) | Farmer/Fisher/  Seaman | 1119 (7.5) | 500 (28.2) | 721 (24.5) | 726 (21.9) | 526 (22.8) | 571 (26.6) | 312 (36.2) | 386 (31.6) | 312 (49) | 466 | 305 | 5944 (27.64) |
|  |  |  |  |  |  |  |  |  |  |  |  |  |  |
| Tertiary sector (Services) | Cadre | 0 (0) | 0 (0) | 0 (0) | 0 (0) | 0 (0) | 0 (0) | 0 (0) | 0 (0) | 0 (0) | 5 (60) | 3 (100) | 8 (75) |
|  | Houseworker | 17 (29.4) | 9 (66.7) | 13 (46.2) | 11 (54.6) | 14 (50) | 15 (40) | 25 (60) | 11 (91) | 38 (73.7) | 24 (70.8) | 20 (65) | 197 (60.4) |
|  | Medical staff/Nurse | 0 (0) | 0 (0) | 0 (0) | 0 (0) | 0 (0) | 0 (0) | 0 (0) | 0 (0) | 0 (0) | 2 (100) | 2 (100) | 4 (100) |
|  | Student/Teacher | 246 (6.9) | 115 (17.4) | 135 (14.8) | 99 (26.3) | 76 (17.1) | 48 (33.3) | 29 (13.8) | 28 (17.9) | 16 (50) | 17 (70.6) | 17 (35.3) | 826 (17.8) |
|  | Commercial service | 0 (0) | 1 (100) | 0 (0) | 0 (0) | 0 (0) | 0 (0) | 0 (0) | 0 (0) | 0 (0) | 8 (87.5) | 14 (85.7) | 23 (86.9) |
|  | *Sub-Total* | 263 (5.3) | 125 (42.4) | 148 (35.8) | 110 (44.5) | 90 (15.6) | 63 (79.4) | 54 (72.2) | 39 (97.4) | 54 (68.5) | 56 (16.1) | 56 (19.6) | 1058 (28) |
|  |  |  |  |  |  |  |  |  |  |  |  |  |  |
|  |  |  |  |  |  |  |  |  |  |  |  |  |  |
| Undefined | Other | 65 (21.5) | 79 (67.1) | 68 (77.9) | 78 (62.8) | 32 (43.7) | 74 (67.6) | 48 (81.3) | 50 (76) | 54 (68.5) | 14 (64.3) | 18 (61.1) | 580 (63.3) |
|  | Not working/Retiree | 18 (0) | 13 (46.1) | 21 (52.4) | 16 (56.3) | 10 (40) | 10 (50) | 9 (77.8) | 12 (58.3) | 20 (65) | 20 (75) | 32 (62.5) | 181 (53.6) |
|  | *Sub-total* | 83 (16.9) | 92 (64.1) | 89 (71.9) | 94 (61.7) | 42 (42.9) | 84 (65.5) | 57 (80.7) | 62 (72.6) | 74 (67.6) | 34 (71) | 50 (62) | 761 (61) |
|  |  |  |  |  |  |  |  |  |  |  |  |  |  |
|  |  |  |  |  |  |  |  |  |  |  |  |  |  |
|  | **Total** | 1465 (8.2) | 717 (31.7) | 958 (27.9) | 930 (26.8) | 658 (24) | 718 (31.9) | 423 (42.1) | 487 (37.4) | 440 (54.3) | 556 (57.7) | 411 (56.7) | 7763 (31) |

**Table S3. Case fatality-rates (CFR) of leptospirosis across two regions in China, 2005-2015**

| **Region** | **Province** | **Case fatality-rates (%)** | | | | | | | | | | | **Annual**  **CFR** | **95% CI** | |
| --- | --- | --- | --- | --- | --- | --- | --- | --- | --- | --- | --- | --- | --- | --- | --- |
|  |  | **2005** | **2006** | **2007** | **2008** | **2009** | **2010** | **2011** | **2012** | **2013** | **2014** | **2015** |  | **Lower** | **Upper** |
| A | Guangdong | 4.69 | 2.99 | 6.06 | 5.95 | 1.69 | 0.00 | 1.75 | 0.00 | 1.92 | 0.00 | 0.00 | 2.28 | 0.88 | 3.67 |
|  | Guangxi | 12.50 | 0.08 | 0.06 | 0.02 | 0.04 | 0.02 | 0.00 | 0.03 | 0.00 | 0.04 | 0.00 | 1.16 | -1.06 | 3.38 |
|  | Hainan | 0.00 | 0.00 | 0.00 | 0.00 | 0.00 | 0.00 | 0.00 | 0.00 | 0.00 | 0.00 | 0.00 | 0.00 | 0.00 | 0.00 |
|  | *CFR by region* | 8.39 | 5.15 | 6.02 | 4.07 | 3.05 | 0.83 | 1.12 | 1.16 | 1.27 | 1.56 | 0.00 | 2.97 | 1.41 | 4.52 |
|  |  |  |  |  |  |  |  |  |  |  |  |  |  |  |  |
|  |  |  |  |  |  |  |  |  |  |  |  |  |  |  |  |
| B | Jiangsu | 0.00 | 0.00 | 14.29 | 0.00 | 0.00 | 0.00 | 0.00 | 0.00 | 0.00 | 0.00 | 0.00 | 1.30 | -1.25 | 3.84 |
|  | Zhejiang | 14.29 | 0.00 | 0.00 | 0.00 | 0.00 | 0.00 | 0.00 | 0.00 | 0.00 | 0.14 | 0.00 | 1.31 | -1.23 | 3.85 |
|  | Anhui | 4.00 | 5.56 | 1.79 | 0.00 | 0.00 | 0.00 | 0.00 | 0.00 | 0.00 | 0.00 | 0.00 | 1.03 | -0.13 | 2.19 |
|  | Fujian | 0.00 | 0.00 | 0.00 | 4.55 | 1.89 | 0.00 | 0.00 | 0.00 | 1.69 | 0.00 | 0.00 | 0.74 | -0.12 | 1.60 |
|  | Jiangxi | 3.37 | 1.85 | 5.17 | 1.64 | 2.13 | 3.70 | 0.00 | 0.00 | 0.00 | 0.00 | 0.00 | 1.62 | 0.54 | 2.71 |
|  | Henan | 0.00 | 0.00 | 0.00 | 0.00 | 0.00 | 0.00 | 0.00 | 0.00 | 0.00 | 0.00 | 0.00 | 0.00 | 0.00 | 0.00 |
|  | Hubei | 10.53 | 4.55 | 1.85 | 2.06 | 5.88 | 0.00 | 0.00 | 0.00 | 0.00 | 0.00 | 7.69 | 2.96 | 0.78 | 5.14 |
|  | Hunan | 8.11 | 3.26 | 7.33 | 2.53 | 2.56 | 2.44 | 3.03 | 4.88 | 0.00 | 0.00 | 0.00 | 3.10 | 1.47 | 4.73 |
|  | Chongqing | 5.36 | 0.00 | 0.00 | 0.00 | 0.00 | 0.00 | 0.00 | 0.00 | 0.00 | 0.00 | 0.00 | 0.49 | -0.47 | 1.44 |
|  | Sichuan | 0.76 | 0.00 | 0.92 | 1.27 | 1.48 | 3.14 | 2.74 | 0.00 | 0.00 | 1.85 | 0.00 | 1.10 | 0.44 | 1.77 |
|  | Guizhou | 17.86 | 11.11 | 23.81 | 6.45 | 6.67 | 0.00 | 7.14 | 6.67 | 42.86 | 25.00 | 0.00 | 13.41 | 5.77 | 21.06 |
|  | Yunnan | 0.88 | 0.77 | 0.00 | 0.00 | 0.00 | 0.00 | 0.00 | 0.00 | 0.00 | 0.00 | 0.00 | 0.15 | -0.05 | 0.35 |
|  | *CFR by region* | 2.75 | 2.11 | 3.51 | 1.59 | 1.53 | 1.68 | 1.21 | 0.99 | 1.14 | 1.04 | 0.27 | 1.62 | 1.09 | 2.15 |

Note: No death cases reported from region C and D.

**Table S4. Temporal distribution of leptospirosis mortality in two regions in China, by province, 2005-2015**

| **Region** | **Province** | **Number of reported death (per 100,000 population)** | | | | | | | | | | | **Total** | **Annual**  **Mortality Rate** |
| --- | --- | --- | --- | --- | --- | --- | --- | --- | --- | --- | --- | --- | --- | --- |
|  |  | **2005** | **2006** | **2007** | **2008** | **2009** | **2010** | **2011** | **2012** | **2013** | **2014** | **2015** |  |  |
| **A** | Guangdong | 3 (0.03) | 2 (0.02) | 4 (0.04) | 5 (0.05) | 1 (0.00) | 0 (0.00) | 1 (0.01) | 0 (0.00) | 1 (0.01) | 0 (0.00) | 0 (0.00) | 17 | 0.01 |
|  | Guangxi | 9 (0.19) | 5 (0.11) | 4 (0.08) | 2 (0.04) | 3 (0.06) | 1 (0.02) | 0 (0.00) | 1 (0.02) | 0 (0.00) | 1 (0.00) | 0 (0.00) | 26 | 0.05 |
|  | *Total* | 12 (0.08) | 7 (0.05) | 8 (0.05) | 7 (0.03) | 4 (0.01) | 1 (0.01) | 1 (0.01) | 1 (0.01) | 1 (0.01) | 1 (0.01) | 0 (0.00) | 43 | 0.02 |
|  |  |  |  |  |  |  |  |  |  |  |  |  |  |  |
| **B** | Jiangsu | 0 (0.00) | 0 (0.00) | 1 (0.01) | 0 (0.00) | 0 (0.00) | 0 (0.00) | 0 (0.00) | 0 (0.00) | 0 (0.00) | 0 (0.00) | 0 (0.00) | 1 | 0.001 |
|  | Zhejiang | 2 (0.04) | 0 (0.00) | 0 (0.00) | 0 (0.00) | 0 (0.00) | 0 (0.00) | 0 (0.00) | 0 (0.00) | 0 (0.00) | 1 (0.02) | 0 (0.00) | 3 | 0.005 |
|  | Anhui | 1 (0.02) | 1 (0.02) | 1 (0.02) | 0 (0.00) | 0 (0.00) | 0 (0.00) | 0 (0.00) | 0 (0.00) | 0 (0.00) | 0 (0.00) | 0 (0.00) | 3 | 0.005 |
|  | Fujian | 0 (0.00) | 0 (0.00) | 0 (0.00) | 2 (0.05) | 1 (0.03) | 0 (0.00) | 0 (0.00) | 0 (0.00) | 1 (0.03) | 0 (0.00) | 0 (0.00) | 4 | 0.01 |
|  | Jiangxi | 3 (0.07) | 1 (0.02) | 3 (0.07) | 1 (0.02) | 1 (0.02) | 1 (0.02) | 0 (0.00) | 0 (0.00) | 0 (0.00) | 0 (0.00) | 0 (0.00) | 10 | 0.02 |
|  | Hubei | 4 (0.07) | 1 (0.02) | 1 (0.02) | 2 (0.03) | 1 (0.02) | 0 (0.00) | 0 (0.00) | 0 (0.00) | 0 (0.00) | 0 (0.00) | 1 (0.02) | 10 | 0.02 |
|  | Hunan | 6 (0.09) | 3 (0.04) | 11 (0.16) | 2 (0.03) | 1 (0.01) | 1 (0.01) | 1 (0.01) | 2 (0.03) | 0 (0.00) | 0 (0.00) | 0 (0.00) | 27 | 0.04 |
|  | Chongqing | 3 (0.10) | 0 (0.00) | 0 (0.00) | 0 (0.00) | 0 (0.00) | 0 (0.00) | 0 (0.00) | 0 (0.00) | 0 (0.00) | 0 (0.00) | 0 (0.00) | 3 | 0.01 |
|  | Sichuan | 6 (0.07) | 0 (0.00) | 2 (0.02) | 3 (0.04) | 3 (0.04) | 8 (0.10) | 2 (0.02) | 0 (0.00) | 0 (0.00) | 2 (0.02) | 0 (0.00) | 26 | 0.03 |
|  | Guizhou | 10 (0.29) | 5 (0.14) | 10 (0.29) | 2 (0.03) | 1 (0.03) | 0 (0.00) | 1 (0.03) | 2 (0.06) | 3 (0.09) | 2 (0.06) | 0 (0.00) | 36 | 0.09 |
|  | Yunnan | 2 (0.04) | 0 (0.00) | 0 (0.00) | 0 (0.00) | 0 (0.00) | 0 (0.00) | 0 (0.00) | 0 (0.00) | 0 (0.00) | 0 (0.00) | 0 (0.00) | 2 | 0.004 |
|  | *Total* | 37 (0.06) | 11 (0.02) | 29 (0.05) | 12 (0.01) | 8 (0.01) | 10 (0.02) | 4 (0.01) | 4 (0.00) | 4 (0.01) | 5 (0.01) | 1 (0.00) | 125 | 0.02 |
| **National Mortality** | | **49 (0.004)** | **18 (0.001)** | **37 (0.003)** | **19 (0.002)** | **12 (0.001)** | **11 (0.001)** | **5 (0.00)** | **5 (0.00)** | **5 (0.00)** | **6 (0.00)** | **1 (0.00)** | **168** | **0.001** |

Note: No death cases reported from region C and D

**Table S5. Number of counties reported leptospirosis each year and new counties that reported leptospirosis during 2005-2010 and 2011-2015 in China**

| **Region** | **Province** | **No. of county reported** | | | | | | | | | | |  |  |  | **No. of new counties** | |
| --- | --- | --- | --- | --- | --- | --- | --- | --- | --- | --- | --- | --- | --- | --- | --- | --- | --- |
|  |  | **2005** | **2006** | **2007** | **2008** | **2009** | **2010** | **2011** | **2012** | **2013** | **2014** | **2015** | **t** | **P value*** |  | **2005-2010** | **2011-2015** |
| **A** | Guangdong | 29 | 32 | 37 | 46 | 33 | 40 | 31 | 27 | 31 | 23 | 25 | 2.792 | .021 |  | 31 | 11 |
|  | Guangxi | 35 | 38 | 39 | 37 | 35 | 32 | 20 | 26 | 16 | 22 | 15 | 7.557 | .000 |  | 38 | 7 |
|  | Hainan | 5 | 4 | 4 | 3 | 3 | 1 | 4 | 5 | 3 | 4 | 1 | -.077 | .941 |  | 5 | 4 |
|  | Total | 69 | 74 | 80 | 86 | 71 | 73 | 55 | 58 | 50 | 49 | 41 |  |  |  | 74 | 22 |
|  |  |  |  |  |  |  |  |  |  |  |  |  |  |  |  |  |  |
| **B** | Jiangsu | 9 | 6 | 7 | 5 | 1 | 1 | 4 | 0 | 0 | 0 | 0 | 2.467 | .036 |  | 21 | 2 |
|  | Zhejiang | 8 | 6 | 11 | 7 | 6 | 11 | 6 | 2 | 4 | 7 | 7 | 2.176 | .058 |  | 14 | 5 |
|  | Anhui | 8 | 8 | 10 | 11 | 8 | 9 | 9 | 6 | 5 | 8 | 6 | 2.514 | .033 |  | 16 | 5 |
|  | Fujian | 18 | 17 | 19 | 24 | 15 | 23 | 20 | 16 | 29 | 24 | 26 | -1.413 | .191 |  | 13 | 8 |
|  | Jiangxi | 24 | 21 | 27 | 22 | 23 | 13 | 16 | 10 | 12 | 8 | 2 | 4.044 | .003 |  | 24 | 5 |
|  | Henan | 0 | 3 | 0 | 0 | 0 | 0 | 0 | 0 | 0 | 0 | 0 | .905 | .389 |  | 3 | 0 |
|  | Hubei | 15 | 11 | 13 | 13 | 9 | 6 | 8 | 6 | 3 | 2 | 6 | 3.485 | .007 |  | 18 | 2 |
|  | Hunan | 29 | 40 | 49 | 32 | 23 | 28 | 18 | 23 | 17 | 18 | 21 | 3.220 | .010 |  | 40 | 16 |
|  | Chongqing | 20 | 10 | 9 | 13 | 11 | 10 | 6 | 7 | 5 | 6 | 4 | 3.467 | .007 |  | 16 | 5 |
|  | Sichuan | 69 | 43 | 46 | 40 | 34 | 38 | 20 | 28 | 26 | 30 | 27 | 3.228 | .010 |  | 37 | 9 |
|  | Guizhou | 23 | 15 | 16 | 14 | 8 | 14 | 7 | 13 | 5 | 5 | 11 | 2.593 | .029 |  | 20 | 6 |
|  | Yunnan | 12 | 9 | 9 | 8 | 9 | 8 | 8 | 8 | 8 | 14 | 11 | -.498 | .630 |  | 12 | 12 |
|  | Total | 235 | 189 | 216 | 189 | 147 | 161 | 122 | 119 | 114 | 122 | 121 |  |  |  | 234 | 75 |
|  |  |  |  |  |  |  |  |  |  |  |  |  |  |  |  |  |  |
| **C** | Beijing | 0 | 0 | 0 | 1 | 1 | 0 | 0 | 0 | 0 | 0 | 0 | 1.430 | .186 |  | 2 | 0 |
|  | Hebei | 0 | 0 | 0 | 0 | 0 | 0 | 1 | 0 | 0 | 1 | 1 | -2.714 | .024 |  | 0 | 3 |
|  | Shanxi | 1 | 0 | 2 | 0 | 0 | 0 | 0 | 0 | 0 | 0 | 0 | 1.324 | .218 |  | 3 | 0 |
|  | Inner Mongolia | 0 | 0 | 0 | 0 | 0 | 0 | 0 | 1 | 0 | 0 | 0 | -1.108 | .297 |  | 0 | 1 |
|  | Liaoning | 1 | 0 | 0 | 0 | 0 | 0 | 0 | 0 | 0 | 0 | 0 | .905 | .389 |  | 1 | 0 |
|  | Jilin | 0 | 0 | 0 | 0 | 0 | 0 | 2 | 0 | 0 | 0 | 0 | -1.108 | .297 |  | 0 | 2 |
|  | Shaanxi | 0 | 0 | 1 | 0 | 2 | 0 | 0 | 0 | 1 | 0 | 0 | .717 | .492 |  | 3 | 1 |
|  | Shandong | 1 | 2 | 0 | 0 | 1 | 5 | 2 | 1 | 0 | 0 | 0 | .980 | .353 |  | 5 | 6 |
|  | Total | 3 | 2 | 3 | 1 | 4 | 5 | 5 | 2 | 1 | 1 | 1 |  |  |  | 14 | 13 |
|  |  |  |  |  |  |  |  |  |  |  |  |  |  |  |  |  |  |
| **D** | Gansu | 0 | 0 | 0 | 0 | 0 | 0 | 0 | 0 | 0 | 1 | 0 | -1.108 | .297 |  | 0 | 1 |
|  | Qinghai | 0 | 0 | 0 | 1 | 0 | 0 | 0 | 0 | 0 | 0 | 0 | .905 | .389 |  | 1 | 0 |
|  | Xinjiang | 0 | 0 | 0 | 1 | 0 | 0 | 0 | 1 | 0 | 0 | 0 | -.129 | .900 |  | 1 | 1 |
|  | Total | 0 | 0 | 0 | 2 | 0 | 0 | 0 | 1 | 0 | 1 | 0 |  |  |  | 2 | 2 |
| **TOTAL (all region)** | | 307 | 265 | 299 | 278 | 222 | 239 | 182 | 180 | 165 | 173 | 163 | 6.206 | .000 |  | 324 | 112 |

*test of average number of counties reported between two blocks of period: 2005-2010 and 2011-2015

**Table S6. Disability-adjusted life-years (DALY) estimates of leptospirosis in China, by gender, by age and year**

| **Characteristics** | | **DALYs estimate** | | | | | | | | | | | **Total** | **Annual DALY** |
| --- | --- | --- | --- | --- | --- | --- | --- | --- | --- | --- | --- | --- | --- | --- |
|  |  | **2005** | **2006** | **2007** | **2008** | **2009** | **2010** | **2011** | **2012** | **2013** | **2014** | **2015** |  |  |
| **Female** | 0-9 | 0.71 | 1.90 | 81.92 | 0.71 | 1.42 | 0.47 | 0.47 | 0.00 | 0.00 | 0.71 | 0.47 | 88.81 | 8.07 |
|  | 10-19 | 157.45 | 220.72 | 440.97 | 71.73 | 75.66 | 2.85 | 1.19 | 143.13 | 0.71 | 1.42 | 1.66 | 1,117.51 | 101.59 |
|  | 20-29 | 192.84 | 201.93 | 72.57 | 68.89 | 67.94 | 3.56 | 3.09 | 3.32 | 3.80 | 8.55 | 3.32 | 629.83 | 57.26 |
|  | 30-39 | 185.59 | 11.87 | 66.89 | 61.46 | 9.26 | 9.02 | 4.99 | 4.75 | 5.70 | 7.60 | 6.89 | 374.03 | 34.00 |
|  | 40-49 | 21.14 | 10.69 | 107.78 | 58.51 | 88.92 | 53.25 | 9.26 | 10.45 | 10.45 | 9.97 | 7.12 | 387.55 | 35.23 |
|  | 50-59 | 153.33 | 9.50 | 82.86 | 51.35 | 12.11 | 14.49 | 6.65 | 41.91 | 8.31 | 9.97 | 6.41 | 396.90 | 36.08 |
|  | 60-69 | 29.42 | 3.09 | 4.51 | 6.41 | 25.47 | 36.65 | 4.27 | 5.46 | 5.22 | 7.84 | 4.27 | 132.63 | 12.06 |
|  | 70-79 | 0.47 | 0.71 | 1.19 | 2.14 | 18.23 | 1.66 | 1.42 | 2.14 | 1.66 | 2.61 | 1.19 | 33.43 | 3.04 |
|  | 80-89 | 0.00 | 0.00 | 0.24 | 0.47 | 0.00 | 0.00 | 0.00 | 0.24 | 0.47 | 0.47 | 0.47 | 2.37 | 0.22 |
|  | 90+ | 0.00 | 0.00 | 0.24 | 0.00 | 0.00 | 0.00 | 0.00 | 0.00 | 0.00 | 0.00 | 0.24 | 0.47 | 0.04 |
|  | **Total** | 740.96 | 460.41 | 859.17 | 321.69 | 299.03 | 121.96 | 31.35 | 211.41 | 36.34 | 49.16 | 32.06 | 3,163.54 | 287.59 |
|  |  |  |  |  |  |  |  |  |  |  |  |  |  |  |
| **Male** | 0-9 | 90.09 | 4.75 | 83.30 | 82.11 | 2.85 | 1.90 | 1.42 | 0.95 | 1.19 | 0.71 | 0.24 | 269.51 | 24.50 |
|  | 10-19 | 839.13 | 306.58 | 377.76 | 94.99 | 16.15 | 9.50 | 153.83 | 75.30 | 78.93 | 4.27 | 3.56 | 1,960.01 | 178.18 |
|  | 20-29 | 587.81 | 142.38 | 149.34 | 22.80 | 185.79 | 69.59 | 6.41 | 66.27 | 124.47 | 70.51 | 7.36 | 1,432.75 | 130.25 |
|  | 30-39 | 261.28 | 75.65 | 276.79 | 181.31 | 17.34 | 22.09 | 10.69 | 11.16 | 6.89 | 167.17 | 8.07 | 1,038.44 | 94.40 |
|  | 40-49 | 206.24 | 62.27 | 189.03 | 67.50 | 62.04 | 26.12 | 92.72 | 19.47 | 14.72 | 63.46 | 57.04 | 860.63 | 78.24 |
|  | 50-59 | 172.58 | 121.69 | 229.99 | 133.90 | 56.36 | 117.17 | 17.34 | 14.25 | 13.54 | 46.75 | 16.15 | 939.71 | 85.43 |
|  | 60-69 | 64.25 | 35.31 | 40.92 | 85.95 | 40.86 | 141.29 | 31.01 | 14.49 | 66.11 | 12.11 | 15.44 | 547.75 | 49.80 |
|  | 70-79 | 3.32 | 20.76 | 4.27 | 39.17 | 3.56 | 4.75 | 3.32 | 5.46 | 3.80 | 2.61 | 4.75 | 95.80 | 8.71 |
|  | 80-89 | 0.24 | 0.00 | 1.19 | 0.47 | 0.24 | 0.00 | 0.71 | 0.47 | 0.47 | 0.71 | 0.24 | 4.75 | 0.43 |
|  | 90+ | 0.00 | 0.00 | 0.00 | 0.00 | 0.00 | 0.00 | 0.00 | 0.00 | 0.24 | 0.00 | 0.00 | 0.24 | 0.02 |
|  | **Total** | 2,224.94 | 769.39 | 1352.60 | 708.21 | 385.19 | 392.42 | 317.47 | 207.83 | 310.37 | 368.33 | 112.85 | 7,149.59 | 649.96 |
|  | |  |  |  |  |  |  |  |  |  |  |  |  |  |
| **DALYs (both sexes)** | | 2,965.90 | 1,229.80 | 2,211.77 | 1,029.91 | 684.22 | 514.37 | 348.82 | 419.24 | 346.71 | 417.49 | 144.91 | 10,313.13 | 937.56 |

**Table S7. Years of life lost (YLL) estimates for leptospirosis in China, by gender, by age and year**

| **Characteristics** | | **YLLs** | | | | | | | | | | | **Total** | **Annual YLLs** |
| --- | --- | --- | --- | --- | --- | --- | --- | --- | --- | --- | --- | --- | --- | --- |
|  |  | **2005** | **2006** | **2007** | **2008** | **2009** | **2010** | **2011** | **2012** | **2013** | **2014** | **2015** |  |  |
| **Female** | 0-9 | 0.00 | 0.00 | 79.31 | 0.00 | 0.00 | 0.00 | 0.00 | 0.00 | 0.00 | 0.00 | 0.00 | 79.31 | 7.21 |
|  | 10-19 | 143.68 | 215.02 | 435.03 | 69.36 | 72.34 | 0.00 | 0.00 | 141.71 | 0.00 | 0.00 | 0.00 | 1,077.14 | 97.92 |
|  | 20-29 | 177.40 | 196.23 | 66.40 | 63.43 | 63.43 | 0.00 | 0.00 | 0.00 | 0.00 | 0.00 | 0.00 | 566.89 | 51.54 |
|  | 30-39 | 155.90 | 0.00 | 52.17 | 48.64 | 0.00 | 0.00 | 0.00 | 0.00 | 0.00 | 0.00 | 0.00 | 256.71 | 23.34 |
|  | 40-49 | 0.00 | 0.00 | 88.54 | 43.79 | 80.85 | 41.85 | 0.00 | 0.00 | 0.00 | 0.00 | 0.00 | 255.03 | 23.18 |
|  | 50-59 | 138.13 | 0.00 | 68.61 | 37.10 | 0.00 | 0.00 | 0.00 | 33.36 | 0.00 | 0.00 | 0.00 | 277.20 | 25.20 |
|  | 60-69 | 25.15 | 0.00 | 0.00 | 0.00 | 20.72 | 27.86 | 0.00 | 0.00 | 0.00 | 0.00 | 0.00 | 73.73 | 6.70 |
|  | 70-79 | 0.00 | 0.00 | 0.00 | 0.00 | 17.28 | 0.00 | 0.00 | 0.00 | 0.00 | 0.00 | 0.00 | 17.28 | 1.57 |
|  | 80-89 | 0.00 | 0.00 | 0.00 | 0.00 | 0.00 | 0.00 | 0.00 | 0.00 | 0.00 | 0.00 | 0.00 | 0.00 | 0.00 |
|  | 90+ | 0.00 | 0.00 | 0.00 | 0.00 | 0.00 | 0.00 | 0.00 | 0.00 | 0.00 | 0.00 | 0.00 | 0.00 | 0.00 |
|  | **Total** | 640.26 | 411.25 | 790.06 | 262.32 | 254.62 | 69.71 | 0.00 | 175.07 | 0.00 | 0.00 | 0.00 | 2,603.29 | 236.66 |
|  |  |  |  |  |  |  |  |  |  |  |  |  |  |  |
|  |  |  |  |  |  |  |  |  |  |  |  |  |  |  |
| **Male** | 0-9 | 81.30 | 0.00 | 78.31 | 78.31 | 0.00 | 0.00 | 0.00 | 0.00 | 0.00 | 0.00 | 0.00 | 237.92 | 21.63 |
|  | 10-19 | 788.78 | 286.39 | 353.77 | 74.33 | 0.00 | 0.00 | 147.66 | 69.36 | 76.32 | 0.00 | 0.00 | 1,796.61 | 163.33 |
|  | 20-29 | 550.05 | 128.84 | 130.82 | 0.00 | 174.63 | 58.67 | 0.00 | 58.67 | 117.35 | 62.44 | 0.00 | 1,281.47 | 116.50 |
|  | 30-39 | 210.22 | 54.51 | 251.62 | 153.76 | 0.00 | 0.00 | 0.00 | 0.00 | 0.00 | 154.35 | 0.00 | 824.46 | 74.95 |
|  | 40-49 | 174.18 | 39.95 | 161.72 | 39.95 | 40.90 | 0.00 | 78.95 | 0.00 | 0.00 | 39.95 | 44.69 | 620.29 | 56.39 |
|  | 50-59 | 138.14 | 101.03 | 197.45 | 99.23 | 36.17 | 92.71 | 0.00 | 0.00 | 0.00 | 30.60 | 0.00 | 695.33 | 63.21 |
|  | 60-69 | 48.58 | 26.05 | 27.86 | 70.99 | 24.24 | 124.90 | 22.46 | 0.00 | 52.10 | 0.00 | 0.00 | 397.18 | 36.11 |
|  | 70-79 | 0.00 | 18.15 | 0.00 | 34.66 | 0.00 | 0.00 | 0.00 | 0.00 | 0.00 | 0.00 | 0.00 | 52.81 | 4.80 |
|  | 80-89 | 0.00 | 0.00 | 0.00 | 0.00 | 0.00 | 0.00 | 0.00 | 0.00 | 0.00 | 0.00 | 0.00 | 0.00 | 0.00 |
|  | 90+ | 0.00 | 0.00 | 0.00 | 0.00 | 0.00 | 0.00 | 0.00 | 0.00 | 0.00 | 0.00 | 0.00 | 0.00 | 0.00 |
|  | **Total** | 1991.25 | 654.92 | 1201.55 | 551.23 | 275.94 | 276.28 | 249.07 | 128.03 | 245.77 | 287.34 | 44.69 | 5,906.07 | 536.92 |
|  | |  |  |  |  |  |  |  |  |  |  |  |  |  |
| **YLLs (both sexes)** | | 2,631.51 | 1,066.17 | 1,991.61 | 813.55 | 530.56 | 345.99 | 249.07 | 303.10 | 245.77 | 287.34 | 44.69 | 8,509.36 | 773.58 |

**Table S8. Years of life lost (YLLs) estimates for leptospirosis in China, by region, gender and by age group during each block of periods**

| **Region** | **Age group** | **2005-2010** | | | **2011-2015** | | | **2005-2015** | | |
| --- | --- | --- | --- | --- | --- | --- | --- | --- | --- | --- |
|  |  | **Males** | **Females** | **Both sexes** | **Males** | **Females** | **Both sexes** | **Males** | **Females** | **Both sexes** |
| **A** | 0-9 | 0 | 0 | 0 | 0 | 0 | 0 | 0 | 0 | 0 |
|  | 10-19 | 74.33 | 215.02 | 289.35 | 0 | 68.38 | 68.38 | 74.33 | 283.4 | 357.73 |
|  | 20-29 | 484.64 | 123.68 | 608.32 | 61.45 | 0 | 61.45 | 546.09 | 123.68 | 669.77 |
|  | 30-39 | 150.23 | 53.34 | 203.57 | 52.17 | 0 | 52.17 | 202.4 | 53.34 | 255.74 |
|  | 40-49 | 248.32 | 126.54 | 374.86 | 0 | 0 | 0 | 248.32 | 126.54 | 374.86 |
|  | 50-59 | 243.82 | 37.1 | 280.92 | 0 | 0 | 0 | 243.82 | 37.1 | 280.92 |
|  | 60-69 | 21.6 | 0 | 21.6 | 22.46 | 0 | 22.46 | 44.06 | 0 | 44.06 |
|  | 70-79 | 52.81 | 0 | 52.81 | 0 | 0 | 0 | 52.81 | 0 | 52.81 |
|  | 80-89 | 0 | 0 | 0 | 0 | 0 | 0 | 0 | 0 | 0 |
|  | 90+ | 0 | 0 | 0 | 0 | 0 | 0 | 0 | 0 | 0 |
| Total |  | 1,275.75 | 555.68 | 1,831.43 | 136.08 | 68.38 | 204.46 | 1,411.83 | 624.06 | 2,035.89 |
|  |  |  |  |  |  |  |  |  |  |  |
| **B** | 0-9 | 237.92 | 79.31 | 317.23 | 0 | 0 | 0 | 237.92 | 79.31 | 317.23 |
|  | 10-19 | 1,428.94 | 720.41 | 2,149.35 | 293.34 | 73.33 | 366.67 | 1,722.28 | 793.74 | 2,516.02 |
|  | 20-29 | 558.37 | 443.21 | 1,001.58 | 118.34 | 58.67 | 177.01 | 676.71 | 501.88 | 1,178.59 |
|  | 30-39 | 519.88 | 203.37 | 723.25 | 102.18 | 0 | 102.18 | 622.06 | 203.37 | 825.43 |
|  | 40-49 | 208.38 | 128.49 | 336.87 | 163.59 | 0 | 163.59 | 371.97 | 128.49 | 500.46 |
|  | 50-59 | 420.91 | 206.74 | 627.65 | 30.6 | 33.36 | 63.96 | 451.51 | 240.1 | 691.61 |
|  | 60-69 | 301.02 | 73.73 | 374.75 | 52.1 | 0 | 52.1 | 353.12 | 73.73 | 426.85 |
|  | 70-79 | 0 | 17.28 | 17.28 | 0 | 0 | 0 | 0 | 17.28 | 17.28 |
|  | 80-89 | 0 | 0 | 0 | 0 | 0 | 0 | 0 | 0 | 0 |
|  | 90+ | 0 | 0 | 0 | 0 | 0 | 0 | 0 | 0 | 0 |
| Total |  | 3,675.42 | 1,872.54 | 5,547.96 | 760.15 | 165.36 | 925.51 | 4,435.57 | 2,037.9 | 6,473.47 |
| **Total (A+B)** | | **4,951.17** | **2,428.22** | **7,379.39** | **896.23** | **233.74** | **1,129.97** | **5,847.4** | **2,661.96** | **8,509.36** |

**Table S9. Years-lived with disability (YLD) estimates for leptospirosis in China, by gender, by age and year**

| **Characteristics** | | **YLDs** | | | | | | | | | | | **Total** | **Annual YLDs** |
| --- | --- | --- | --- | --- | --- | --- | --- | --- | --- | --- | --- | --- | --- | --- |
|  |  | **2005** | **2006** | **2007** | **2008** | **2009** | **2010** | **2011** | **2012** | **2013** | **2014** | **2015** |  |  |
| **Female** | 0-9 | 0.71 | 1.90 | 2.61 | 0.71 | 1.42 | 0.47 | 0.47 | 0.00 | 0.00 | 0.71 | 0.47 | 9.50 | 0.86 |
|  | 10-19 | 13.77 | 5.70 | 5.94 | 2.37 | 3.32 | 2.85 | 1.19 | 1.42 | 0.71 | 1.42 | 1.66 | 40.37 | 3.67 |
|  | 20-29 | 15.44 | 5.70 | 6.17 | 5.46 | 4.51 | 3.56 | 3.09 | 3.32 | 3.80 | 8.55 | 3.32 | 62.94 | 5.72 |
|  | 30-39 | 29.69 | 11.87 | 14.72 | 12.82 | 9.26 | 9.02 | 4.99 | 4.75 | 5.70 | 7.60 | 6.89 | 117.32 | 10.67 |
|  | 40-49 | 21.14 | 10.69 | 19.24 | 14.72 | 8.07 | 11.40 | 9.26 | 10.45 | 10.45 | 9.97 | 7.12 | 132.52 | 12.05 |
|  | 50-59 | 15.20 | 9.50 | 14.25 | 14.25 | 12.11 | 14.49 | 6.65 | 8.55 | 8.31 | 9.97 | 6.41 | 119.70 | 10.88 |
|  | 60-69 | 4.27 | 3.09 | 4.51 | 6.41 | 4.75 | 8.79 | 4.27 | 5.46 | 5.22 | 7.84 | 4.27 | 58.90 | 5.35 |
|  | 70-79 | 0.47 | 0.71 | 1.19 | 2.14 | 0.95 | 1.66 | 1.42 | 2.14 | 1.66 | 2.61 | 1.19 | 16.15 | 1.47 |
|  | 80-89 | 0.00 | 0.00 | 0.24 | 0.47 | 0.00 | 0.00 | 0.00 | 0.24 | 0.47 | 0.47 | 0.47 | 2.37 | 0.22 |
|  | 90+ | 0.00 | 0.00 | 0.24 | 0.00 | 0.00 | 0.00 | 0.00 | 0.00 | 0.00 | 0.00 | 0.24 | 0.47 | 0.04 |
|  | **Total** | 100.70 | 49.16 | 69.11 | 59.37 | 44.41 | 52.25 | 31.35 | 36.34 | 36.34 | 49.16 | 32.06 | 560.25 | 50.93 |
|  |  |  |  |  |  |  |  |  |  |  |  |  |  |  |
| **Male** | 0-9 | 8.79 | 4.75 | 4.99 | 3.80 | 2.85 | 1.90 | 1.42 | 0.95 | 1.19 | 0.71 | 0.24 | 31.59 | 2.87 |
|  | 10-19 | 50.35 | 20.19 | 23.99 | 20.66 | 16.15 | 9.50 | 6.17 | 5.94 | 2.61 | 4.27 | 3.56 | 163.40 | 14.85 |
|  | 20-29 | 37.76 | 13.54 | 18.52 | 22.80 | 11.16 | 10.92 | 6.41 | 7.60 | 7.12 | 8.07 | 7.36 | 151.28 | 13.75 |
|  | 30-39 | 51.06 | 21.14 | 25.17 | 27.55 | 17.34 | 22.09 | 10.69 | 11.16 | 6.89 | 12.82 | 8.07 | 213.98 | 19.45 |
|  | 40-49 | 32.06 | 22.32 | 27.31 | 27.55 | 21.14 | 26.12 | 13.77 | 19.47 | 14.72 | 23.51 | 12.35 | 240.34 | 21.85 |
|  | 50-59 | 34.44 | 20.66 | 32.54 | 34.67 | 20.19 | 24.46 | 17.34 | 14.25 | 13.54 | 16.15 | 16.15 | 244.38 | 22.22 |
|  | 60-69 | 15.67 | 9.26 | 13.06 | 14.96 | 16.62 | 16.39 | 8.55 | 14.49 | 14.01 | 12.11 | 15.44 | 150.57 | 13.69 |
|  | 70-79 | 3.32 | 2.61 | 4.27 | 4.51 | 3.56 | 4.75 | 3.32 | 5.46 | 3.80 | 2.61 | 4.75 | 42.99 | 3.91 |
|  | 80-89 | 0.24 | 0.00 | 1.19 | 0.47 | 0.24 | 0.00 | 0.71 | 0.47 | 0.47 | 0.71 | 0.24 | 4.75 | 0.43 |
|  | 90+ | 0.00 | 0.00 | 0.00 | 0.00 | 0.00 | 0.00 | 0.00 | 0.00 | 0.24 | 0.00 | 0.00 | 0.24 | 0.02 |
|  | **Total** | 233.69 | 114.47 | 151.05 | 156.98 | 109.25 | 116.14 | 68.40 | 79.80 | 64.60 | 80.99 | 68.16 | 1,243.52 | 113.05 |
|  | |  |  |  |  |  |  |  |  |  |  |  |  |  |
| **YLDs (both sexes)** | | 334.39 | 163.63 | 220.16 | 216.36 | 153.66 | 168.38 | 99.75 | 116.14 | 100.94 | 130.15 | 100.22 | 1,803.77 | 163.98 |

**Table S10. Geographical distribution of YLD, YLL, and DALY estimates during both periods in China**

|  |  | **YLD** | | **TOTAL YLD  (2005-2015)** | **%change** |  | **YLL** | | **Total YLL (2005-2015)** | **%change** |  | **DALY** | | **Total DALY (2005-2015)** | **%change** |
| --- | --- | --- | --- | --- | --- | --- | --- | --- | --- | --- | --- | --- | --- | --- | --- |
|  |  | **2005-2010** | **2011-2015** |  |  |  | **2005-2010** | **2011-2015** |  |  |  | **2005-2010** | **2011-2015** |  |  |
| **Region A** |  |  |  |  |  |  |  |  |  |  |  |  |  |  |  |
| Guangdong |  | 91.44 | 51.54 | 142.97 | -43.64 |  | 629.51 | 83.91 | 713.42 | -86.7 |  | 720.95 | 135.45 | 856.39 | -81.21 |
| Guangxi |  | 91.20 | 31.59 | 122.78 | -65.36 |  | 1201.92 | 120.55 | 1,322.47 | -90.0 |  | 1,293.12 | 152.14 | 1,445.25 | -88.23 |
| Hainan |  | 6.41 | 4.75 | 11.16 | -25.90 |  | 0 | 0 | 0.00 | 0.0 |  | 6.41 | 4.75 | 11.16 | -25.90 |
| Total | | 189.04 | 87.87 | 276.92 | -53.52 |  | 1831.43 | 204.46 | 2,035.89 | -88.8 |  | 2,020.47 | 292.33 | 2,312.81 | -85.53 |
|  |  |  |  |  |  |  |  |  |  |  |  |  |  |  |  |
| **Region B** |  |  |  |  |  |  |  |  |  |  |  |  |  |  |  |
| Jiangsu |  | 7.60 | 0.95 | 8.55 | -87.50 |  | 30.60 | 0 | 30.60 | -100.0 |  | 38.20 | 0.95 | 39.15 | -97.51 |
| Zhejiang |  | 24.94 | 7.12 | 32.06 | -71.43 |  | 69.53 | 30.60 | 100.13 | -56.0 |  | 94.47 | 37.72 | 132.19 | -60.07 |
| Anhui |  | 54.62 | 18.29 | 72.91 | -66.52 |  | 168.19 | 0 | 168.19 | -100.0 |  | 222.81 | 18.29 | 241.10 | -91.79 |
| Fujian |  | 56.76 | 61.51 | 118.27 | 8.37 |  | 106.46 | 26.05 | 132.51 | -75.5 |  | 163.22 | 87.56 | 250.78 | -46.35 |
| Jiangxi |  | 77.42 | 20.19 | 97.61 | -73.93 |  | 487.59 | 0 | 487.59 | -100.0 |  | 565.01 | 20.19 | 585.20 | -96.43 |
| Henan |  | 0.71 | 0.00 | 0.71 | -100.00 |  | 0.00 | 0.00 | 0.00 | 0.0 |  | 0.71 | 0.00 | 0.71 | -100.00 |
| Hubei |  | 56.52 | 11.40 | 67.92 | -79.83 |  | 387.76 | 44.69 | 432.45 | -88.5 |  | 444.28 | 56.09 | 500.37 | -87.38 |
| Hunan |  | 107.11 | 42.27 | 149.38 | -60.53 |  | 1,239.91 | 131.98 | 1,371.89 | -89.4 |  | 1,347.02 | 174.25 | 1,521.27 | -87.06 |
| Chongqing |  | 35.86 | 10.92 | 46.79 | -69.54 |  | 148.15 | 0.00 | 148.15 | -100.0 |  | 184.01 | 10.92 | 194.94 | -94.06 |
| Sichuan |  | 436.52 | 115.90 | 552.41 | -73.45 |  | 900.02 | 213.52 | 1,113.54 | -76.3 |  | 1,336.54 | 329.42 | 1,665.95 | -75.35 |
| Guizhou |  | 42.75 | 17.81 | 60.56 | -58.33 |  | 1,893.39 | 478.67 | 2,372.06 | -74.7 |  | 1,936.14 | 496.48 | 2,432.62 | -74.36 |
| Yunnan |  | 161.97 | 148.20 | 310.17 | -8.50 |  | 116.36 | 0.00 | 116.36 | -100.0 |  | 278.33 | 148.20 | 426.53 | -46.76 |
| Total | | 1,062.79 | 454.57 | 1,517.36 | -57.23 |  | 5,547.96 | 925.51 | 6,473.47 | -83.3 |  | 6,610.75 | 1,380.08 | 7,990.83 | -79.12 |
|  |  |  |  |  |  |  |  |  |  |  |  |  |  |  |  |
| **Region C** |  |  |  |  |  |  |  |  |  |  |  |  |  |  |  |
| Beijing |  | 0.47 | 0.00 | 0.47 | -100.00 |  | 0 | 0 | 0.00 | 0 |  | 0.47 | 0.00 | 0.47 | -100.00 |
| Hebei |  | 0.00 | 0.71 | 0.71 | 71.25 |  | 0 | 0 | 0.00 | 0 |  | 0.00 | 0.71 | 0.71 | 71.25 |
| Shanxi |  | 0.71 | 0.00 | 0.71 | -100.00 |  | 0 | 0 | 0.00 | 0 |  | 0.71 | 0.00 | 0.71 | -100.00 |
| Inner Mongolia | | 0.00 | 0.24 | 0.24 | 24.00 |  | 0 | 0 | 0.00 | 0 |  | 0.00 | 0.24 | 0.24 | 24.00 |
| Liaoning |  | 0.24 | 0.00 | 0.24 | -100.00 |  | 0 | 0 | 0.00 | 0 |  | 0.24 | 0.00 | 0.24 | -100.00 |
| Shandong |  | 2.14 | 2.61 | 4.75 | 22.22 |  | 0 | 0 | 0.00 | 0 |  | 2.14 | 2.61 | 4.75 | 22.22 |
| Jilin |  | 0.00 | 0.47 | 0.47 | 47.50 |  | 0 | 0 | 0.00 | 0 |  | 0.00 | 0.47 | 0.47 | 47.50 |
| Shaanxi |  | 0.71 | 0.24 | 0.95 | -66.67 |  | 0 | 0 | 0.00 | 0 |  | 0.71 | 0.24 | 0.95 | -66.67 |
| Total | | 4.27 | 4.28 | 8.55 | 0.06 |  | 0 | 0 | 0.00 | 0 |  | 4.27 | 4.28 | 8.55 | 0.06 |
|  |  |  |  |  |  |  |  |  |  |  |  |  |  |  |  |
| **Region D** |  |  |  |  |  |  |  |  |  |  |  |  |  |  |  |
| Gansu |  | 0.00 | 0.24 | 0.24 | 23.75 |  | 0 | 0 | 0.00 | 0 |  | 0.00 | 0.24 | 0.24 | 23.75 |
| Qinghai |  | 0.24 | 0.00 | 0.24 | -100.00 |  | 0 | 0 | 0.00 | 0 |  | 0.24 | 0.00 | 0.24 | -100.00 |
| Xinjiang |  | 0.24 | 0.24 | 0.47 | 0.00 |  | 0 | 0 | 0.00 | 0 |  | 0.24 | 0.24 | 0.47 | 0.00 |
| Total | | 0.47 | 0.47 | 0.95 | 0.00 |  | 0 | 0 | 0.00 | 0 |  | 0.47 | 0.47 | 0.95 | 0.00 |
|  |  |  |  |  |  |  |  |  |  |  |  |  |  |  |  |
| **TOTAL (A+B+C+D)** | | 1,256.58 | 547.19 | 1,803.77 | -56.45 |  | 7,379.39 | 1,129.97 | 8,509.36 | -84.7 |  | 8,635.97 | 1,677.16 | 10,313.13 | -80.58 |
